# Innovative Applications of High Hydrostatic Pressure in Winemaking

**DOI:** 10.1111/1541-4337.70204

**Published:** 2025-05-27

**Authors:** Laura Otero, Lucía del Prado, Antonio Morata

**Affiliations:** ^1^ Institute of Food Science, Technology and Nutrition, MALTA‐Consolider Team Spanish National Research Council (ICTAN‐CSIC) Madrid Spain; ^2^ ETSIAAB Universidad Politécnica de Madrid Madrid Spain; ^3^ enotecUPM, MALTA‐Consolider Team, ETSIAAB Universidad Politécnica de Madrid Madrid Spain

## Abstract

Wine industry faces, today, great challenges, including the production of wines with low SO_2_ content, the reduction of winemaking times, or the elaboration of wines with own distinctive characteristics, among others. To assess the potential and opportunities that high hydrostatic pressure (HHP) offers to meet these challenges, an exhaustive bibliographical review has been carried out to compile the scientific studies performed so far on this subject. The studies consulted reveal that high‐pressure processing could be applied at various stages of the winemaking process with different objectives, including reducing the microbial load, accelerating solid–liquid extraction processes, or enhancing chemical changes in wine composition. This would make it possible to reduce SO_2_ levels, shorten vinification times by speeding up certain stages such as must maceration or wine aging, and apply new biotechnologies for wine fermentation capable of producing wines with unique organoleptic profiles. However, the potential of HHP for winemaking has not yet been fully explored, and, thus, based on the observed effects of HHP in food matrices other than wine, this review identifies new opportunities with potential interest. Finally, difficulties associated with HHP implementation in the wine industry are also evaluated to give a rough idea of its industrial feasibility. This review should encourage further research to optimize high‐pressure‐based solutions capable of addressing current challenges of the wine industry.

## 1 Introduction

Wine industry is an economically important sector that, in 2023, generated a total revenue of $328.2 × 10^3^ million worldwide (Statista Market Insights 2024). According to the International Organization of Vine and Wine (OIV), global wine production was 237.3 × 10^6^ hL in that year, whereas consumption was 221 × 10^6^ hL (OIV 2024b). Although production in 2023 was historically low due to extreme climatic conditions and widespread fungal diseases, world wine production has remained fairly stable over the last few years. By contrast, consumption has exhibited a consistent decline since 2018 that can be attributed to a number of factors. These include the reduction in Chinese demand, the COVID‐19 pandemic, the war in Ukraine and the associated energy crisis and supply chain disruptions, or the current inflation that has increased production and distribution costs, while reducing consumer purchasing power. All these factors have contributed to reducing wine consumption in recent years, thus causing a significant increase in market competition.

**FIGURE 1:**
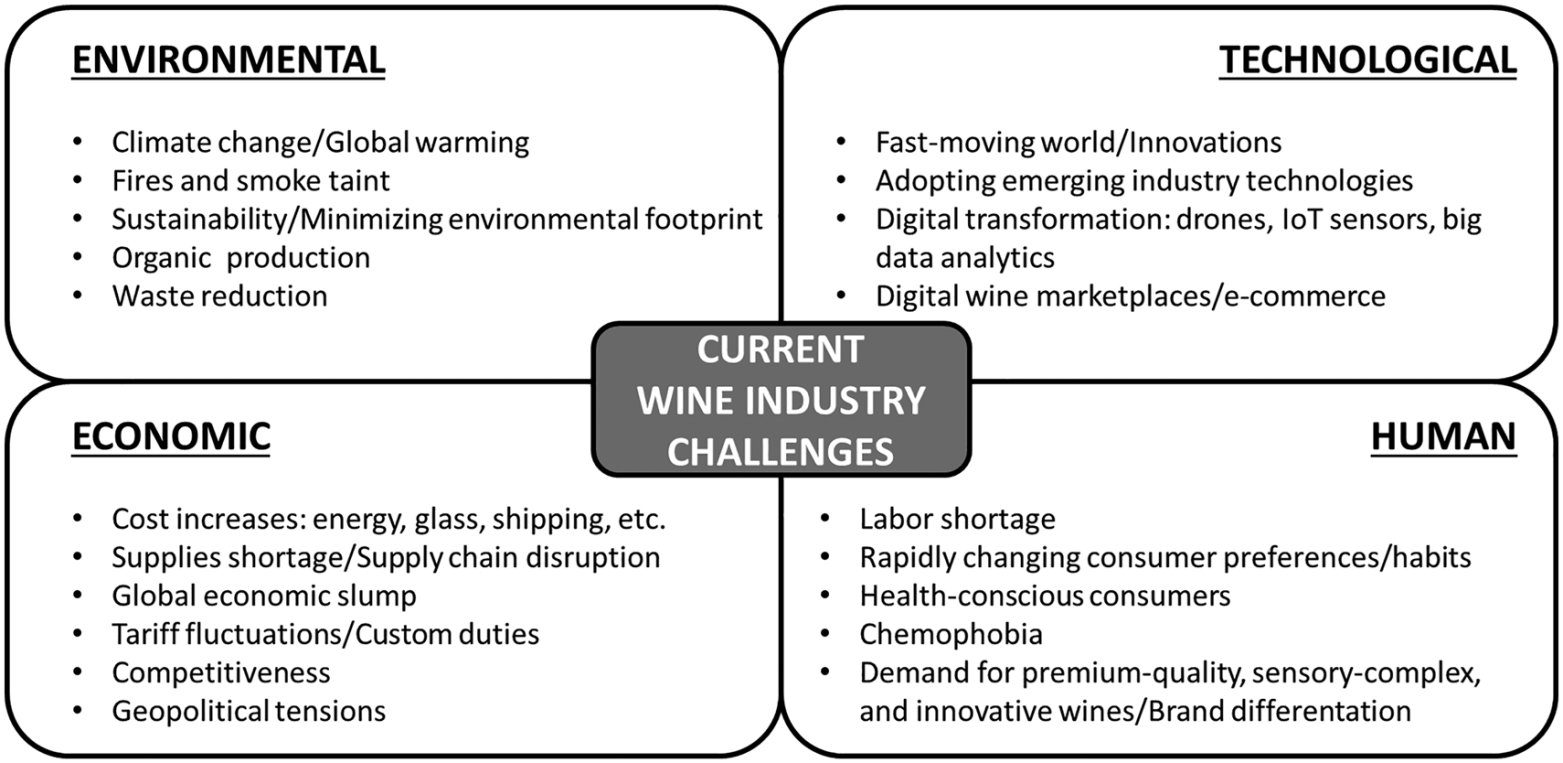
Top challenges facing the wine industry today.

A recent survey among almost 2500 wine experts (producers, intermediaries, and retailers) from more than 30 different countries has revealed that economic topics are perceived as the dominant threats in the wine sector (Loose 2023). The increasing cost of energy and supplies reduces profits and forces producers to raise wine prices and adapt production processes for cost reduction (Figure 1). Geopolitical tensions and conflicts disrupt supply chains, increasing costs for wine production and distribution. Trade barriers, tariffs, and political instability complicate market access and reduce profitability, whereas shifting diplomatic relationships create uncertainty and higher risks for exporters (Carbone 2021). However, the economic challenge is not the only facing the wine industry today, as others related to environmental, technological, and human issues are also perceived as important (Beir 2023; Loose 2023; Sommelier Business 2023; Wagner et al. 2023). Among the environmental challenges, climate change poses a significant threat to the wine industry, impacting grape cultivation and wine production. Rising temperatures not only disrupt the delicate balance among sunshine, temperature, and rainfall that shapes wine characteristics but also intensify wildfires, directly damaging vineyards and affecting wine quality through smoke taint (Beir 2023; Xynas and Barnes 2023). Sustainability is also a key concern for consumers and producers, with eco‐conscious buyers favoring environmentally friendly wine brands (Sánchez‐García et al. 2023; Wagner et al. 2023). On the other hand, technology is nowadays transforming the wine industry, from precision viticulture using drones and data analytics to the rise of e‐commerce and blockchain for traceability. While boosting efficiency and customer experience, these advancements require significant investment, challenging smaller wineries (Luzzani et al. 2021; Tardaguila et al. 2021). Human issues such as growing health concerns, rapidly changing consumer preferences, or chemophobia also have a significant impact on wineries that are constantly evolving to remain competitive (Lombardo et al. 2023; Nieto‐Villegas et al. 2023).

All these economic, environmental, technological, and human factors have dramatically changed the wine market in the last two decades. The international competition, together with the trendy perception of wine as a hedonic good, mainly consumed when socializing and strongly related to culture, self‐identity, and authenticity, has boosted the demand for premium quality, sensory‐complex, and innovative wines with own distinctive characteristics. Moreover, health issues, chemophobia, and the growing demand for organic products have triggered the wine industry's interest in reducing the use of chemical compounds in both vineyard and winery operations (Carbone 2021; Comitini et al. 2017; Nieto‐Villegas et al. 2023; Sánchez‐García et al. 2023). However, some obstacles, such as climate change or the generalized use of standardized industrial practices, do not make it easy to meet the new market demands. Global warming is currently accelerating the maturation of grapes in numerous regions, thus resulting in musts with elevated sugar content and low acidity. Consequently, wines produced from these musts exhibit elevated alcoholic degree, diminished freshness, reduced aging potential, and an increasingly flat sensory profile (Xynas and Barnes 2023). On the other hand, the widespread use of commercial starters of *Saccharomyces cerevisiae* in modern winemaking, while facilitating microbial and technological control of the process, has the disadvantage of yielding wines with similar analytical and sensory properties that hamper brand differentiation (Álvarez et al. 2023; Capece et al. 2019; Comitini et al. 2017). Other consumer requests, such as reducing the use of chemical compounds, are also problematic. For example, avoiding the traditional use of sulfur dioxide in oenology as an antimicrobial, antioxidant, and antioxidasic agent is a challenging undertaking as the implementation of viable alternative strategies capable of effectively substituting it has not yet been achieved (Giacosa et al. 2019; Silva and van Wyk 2021; van Wyk et al. 2018).

In response to all these difficulties in meeting the new market demands, many scientific studies have explored the potential of different technologies to address current challenges in the wine industry (Kumar et al. 2023; Lisanti et al. 2019; Perić et al. 2024). Although the integration of new techniques in such a traditional sector is a challenge in itself, technologies, such as high pressure, electrical pulses, ultrasounds, and pulsed light, have been investigated. Thus, several reviews have evaluated and compared the efficacy of these technologies in improving various aspects of the winemaking process. Some reviews have specifically focused on how these technologies can improve microbial stability and thus reduce the need for SO_2_ addition in wines (Kumar et al. 2023; Lisanti et al. 2019; Morata et al. 2017; Santos et al. 2012; Silva and van Wyk 2021; van Wyk and Silva 2019). Other reviews have evaluated their ability to enhance the extraction of valuable compounds from grape skins and thus accelerate maceration (Morata et al. 2023, 2021, 2019). In addition, several studies have investigated the potential of these technologies to reduce aging times while maintaining or improving wine quality (Krüger et al. 2022; Ma et al. 2022; Morata et al. 2019). As summarized in Table 1, these reviews collectively highlight high hydrostatic pressure (HHP) as one of the most promising technologies currently available for winemaking applications.

**TABLE 1:** Potential and opportunities of non‐thermal technologies to address current challenges in the wine industry.

**Technology Characteristics**		**HHP**	**UHPH**	**PEF**	**US**	**PL**	**β‐IRD**
**Positive effects**	**Potential benefits in winemaking**						
Microbial inactivation	Reduction of SO_2_ doses in wine production Better control of fermentation Easier implementation of new biotechnologies for wine fermentation	YES Vegetative cells	YES Vegetative cells and spores	YES Vegetative cells	Insufficient	YES Vegetative cells and spores	YES Vegetative cells and spores
Inactivation of oxidative enzymes	Reduction of SO_2_ doses in wine production	Insufficient	YES	Insufficient	Insufficient	Insufficient	Insufficient
Enhance solid–liquid extraction	Shortening of the maceration step Shortening of the wood aging step Shortening of the aging on lees step	YES	Only from colloidal particles and fragments in juice lower than 500 µm	YES	YES	Inconclusive results	YES
**Negative effects**							
Degradation of sensory attributes		Minimal	Minimal	Minimal	Flavor and aroma changes	Minimal Color intensity decreases	Minimal Loss of aroma
**Implementation**							
Type of product	Grapes/Crushed grapes	YES	NO	YES	YES	YES	YES
	Must/Wine	YES	YES	YES	YES	YES	YES
Type of process		Discontinuous (Grapes) Semicontinuous (Must/wine)	Continuous	Discontinuous Continuous	Discontinuous Continuous	Continuous	Continuous
Advantages		Compressive technique, low losses of aromatic compounds	Low consumption of water and scarce residues	Short processing time Energy efficiency	Low energy consumption	Cheap Low maintenance	Highly effective at 10 kGy
Limitations		Expensive equipment	Particles in the fluid must be less than 500 µm Expensive equipment	High‐voltage electric pulses pose a safety risk	Not enough to extract and stabilize tannins in the absence of ethanol at the beginning of fermentation	Low penetration depth (< 1 mm)	Penetrability: 6–8 cm Aroma oxidation
Technology used at commercial scale in the food industry		YES	YES	YES	YES	YES	YES
Approved by OIV for enological practices		To improve extraction and control microorganisms	To control microorganisms	To improve extraction (for microbial control under evaluation)	To improve extraction	NO	NO—consumer concerns
**References**		Aganovic et al. (2021), Bañuelos et al. (2016), Corrales et al. (2009), Morata et al. (2015), Morata et al. (2021)	Bañuelos et al. (2020), Loira et al. (2018), Morata and Guamis (2020), Vaquero et al. (2022), Zamora and Guamis (2015)	Bocker and Silva (2022), López et al. (2008), Maza et al. (2020), Puértolas et al. (2010), Puértolas et al. (2011), Vaquero et al. (2021)	Corrales et al. (2008), Natolino and Celotti (2022), Nguyen et al. (2021), Pérez‐Porras et al. (2022), Zhang et al. (2023)	Chakraborty and Parab (2023), Escott et al. (2021, 2017), Santamera et al. (2020)	Błaszak et al. (2019), Morata et al. (2017), Morata, Bañuelos, et al. (2015)

Abbreviations: HHP, high hydrostatic pressure; OIV, International Organization of Vine and Wine; PEF, pulsed electric fields; PL, pulsed light; UHPH, ultra‐high‐pressure homogenization; US, ultrasounds; β‐IRD, β‐irradiation.

HHP is a compressive physical technology that can be applied either to grapes or to wine, in the absence of oxygen, and at low or mild temperature (Table 1). HHP is able to inactivate bacteria and yeasts and can, therefore, be used for wine preservation with reduced amounts of sulfur dioxide (Christofi et al. 2020; Santos et al. 2012; Silva and van Wyk 2021). When applied to grapes, it facilitates the use of some emerging biotechnologies in fermentation (i.e., starters of non*‐Saccharomyces* or yeast‐bacteria co‐inoculations), which have a positive impact on the sensory quality of wine but are sometimes difficult to implant due to competition from the wild microbial population (Bañuelos et al. 2016). Moreover, HHP affects cell wall biopolymers, thus enhancing the extraction of aromatic compounds, tannins, and pigments from grape skins, accelerating the maceration, and improving the wine's color (Morata et al. 2015; Takush and Osborne 2011). Better phenolic extraction, together with the low aeration on HHP processing, also helps to minimize the use of controversial antioxidant additives as sulfites and allows for longer barrel‐aging oxidative processes that enhance the complexity of the aroma. Pressure‐enhanced solid–liquid extraction can also be exploited during wine aging, either with oak wood fragments or on lees, thus shortening this step and improving wine quality (Tao et al. 2016; Valdés et al. 2021).

Previous reviews on the application of HHP technology in winemaking have mainly focused on its effect on the microbial stability of wine and the impact on its chemical and sensorial characteristics (Buzrul 2012; Nunes et al. 2017). This review aims to give a comprehensive view of the true potential of HHP in addressing the current challenges faced by the wine industry. By compiling and analyzing all the studies carried out to date and scattered in the literature, this review identifies different stages of the vinification process where HHP could be applied to reduce SO_2_ levels, shorten vinification times, and obtain wines with unique organoleptic profiles. Furthermore, based on the observed effects of HHP in food matrices other than wine, this review also identifies new opportunities of potential interest to provide a complete picture of the true potential of this technology in winemaking. In addition, the difficulties involved in the implementation of all these innovative applications have also been evaluated to give a rough idea of their industrial feasibility. Therefore, this review should serve as an incentive for further research that makes it possible to exploit the benefits offered by HHP in oenology to the maximum and encourage wineries to adopt this technology.

## 2 HHP Technology

HHP is nowadays a well‐established technology in the food industry with running industrial units in all continents, excluding Antarctica (Houška et al. 2022). In 2020, 1800 million kilos of food were pressure processed worldwide, and this technology generated 18,000 million euros (Hiperbaric 2021). Moreover, thanks to the growing consumer interest in safe, fresh‐like, minimally processed, and additive‐free foods, future perspectives are even more promising, and, thus, the global HHP foods market is expected to grow at a compound annual growth rate of 8.6% between 2024 and 2032 (Expert Market Research 2024).

During HHP processing, foods are immersed in a fluid, typically water, and pressurized, usually at 300–700 MPa, for brief time periods, generally shorter than 10 min. According to the isostatic principle, pressure is transmitted uniformly and instantaneously throughout the product, and, thus, unlike in other processing technologies, processing time does not depend on product size or shape. Moreover, according to Le Chatelier's principle, those chemical reactions, changes in molecular conformation, or phase transitions that involve a volume decrease are enhanced during the HHP treatment, whereas those that involve a volume increase are inhibited (Aganovic et al. 2021; Huang et al. 2017). All these features of HHP food processing represent important advantages over other traditional technologies.

Le Chatelier's principle explains many of the effects of HHP on foods. Since the breaking of covalent bonds implies an increase in molar volume, small, covalently bonded molecules in foods, such as aroma compounds, pigments, or vitamins, are rarely affected by HHP, and, therefore, unlike in thermal processing, the organoleptic and nutritional properties of foods are minimally altered after pressure processing (Oey et al. 2008). By contrast, macromolecules such as proteins and complex carbohydrates, whose molecular structure is maintained by non‐covalent, ionic, or hydrophobic bonds, can undergo significant changes in their native structure after HHP processing because the disruption of these bonds usually involves a reduction in molar volume. In consequence, the properties and functionality of these macromolecules can be significantly affected.

All these effects that pressure causes at the molecular level can be exploited to design a number of interesting applications, some of which have been successfully implemented in the food industry. In this sense, the most widespread application of pressure processing is, undoubtedly, as a method of inactivating microorganisms present in foods, thus extending their shelf life significantly (Rendueles et al. 2011; Sehrawat et al. 2021). Pressure can damage cell membranes, ribosomes, and enzymes, thus affecting cellular processes such as substrate transport, nutrient uptake, or cell division and growth, among others (Abe 2007). By enhancing food safety and extending food shelf life, HHP processing also enables the reduction of synthetic additives used in product formulation, thus helping food processors to satisfy consumers’ demand for clean‐label products (Roobab et al. 2023, 2021). Moreover, HHP processing is considered an environmentally friendly technology, not only because it contributes to reduce food waste but also because it can lead to substantial savings of resources compared to other food processing technologies (Cacace et al. 2020). In this sense, it is important to note that HHP technology only requires electricity to increase pressure and that about 85% of water used in the process is reused, whereas the rest can be collected for other uses, such as cleaning (Hiperbaric 2020b, 2022).

## 3 Applications of HHP in Winemaking

**FIGURE 2:**
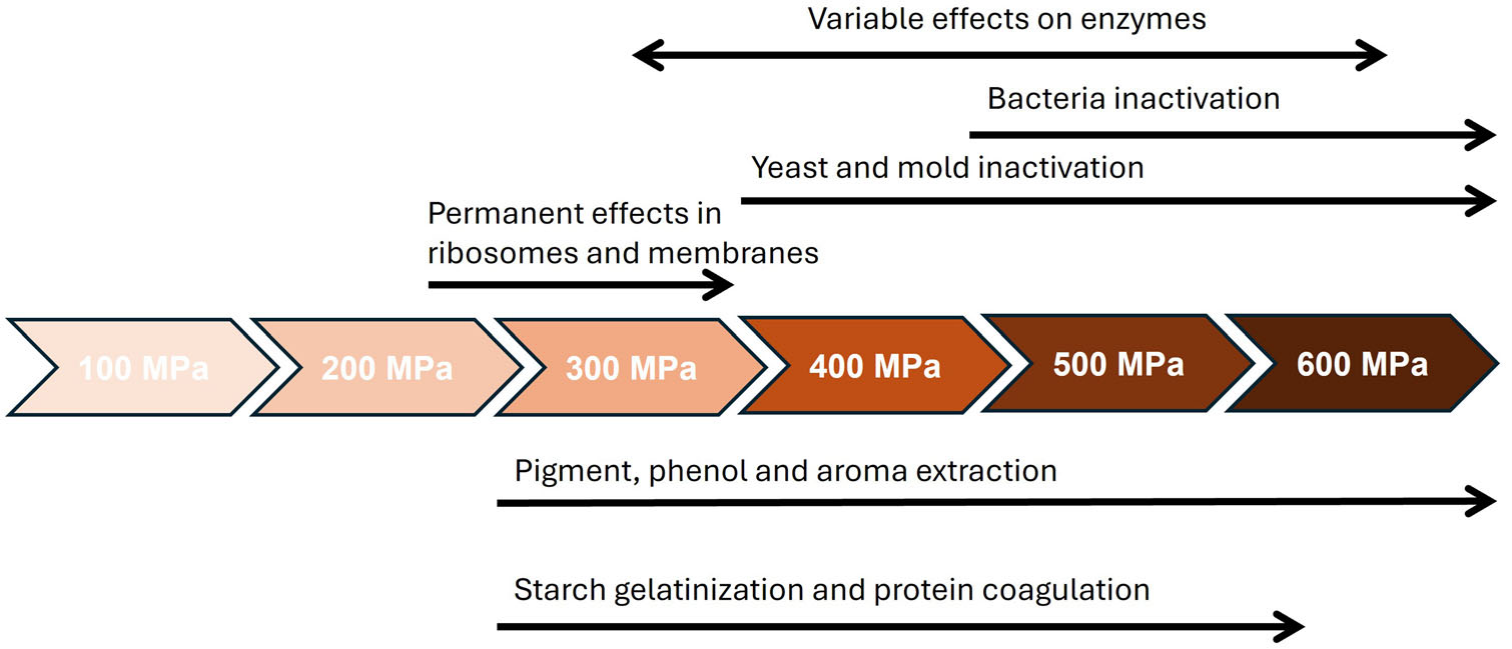
Main effects of pressure that can be exploited in the winemaking process.

Over the last decades, the potential of HHP in the wine sector has been explored by a number of researchers (Christofi et al. 2020; Delfini et al. 1995; Morata et al. 2015, among others). In general, their investigations reveal that, depending on the level applied, HHP has different effects that can be exploited by oenologists to improve the winemaking process. As shown in Figure 2, pressure can damage cell membranes, ribosomes, and enzymes, thus affecting cellular processes and causing the inactivation of microorganisms present in grapes, musts, and wines. In addition, by increasing permeability of membranes, HHP can enhance solid–liquid extraction processes. For these reasons, since 2019, HHP processing, at pressures higher than 150 MPa, is permitted by the OIV to reduce the indigenous microbial load of grapes and musts, limit SO_2_ levels in wine, and accelerate maceration in red winemaking (OIV 2024a).

First studies in the literature were mainly focused on evaluating the potential of HHP processing to prevent microbial spoilage of wine, extend its shelf life, improve safety, and enhance quality (Delfini et al. 1995; Lonvaud‐Funel et al. 1994; Tonello et al. 1998). However, in recent years, many other HHP applications have been envisaged that could be of enormous interest in oenology (Bañuelos et al. 2016; Tao et al. 2016; Valdés et al. 2021). In the following sections, all the opportunities that HHP processing offers to the wine industry are summarized and discussed in detail.

### 3.1 HHP Processing to Control Microbial Population

Many studies reveal that HHP can be applied at different steps of the winemaking process, either on grapes, must, or wine, to reduce their microbial load without any adverse effects on the physicochemical and organoleptic properties of the obtained wine (Delfini et al. 1995; Morata et al. 2015; van Wyk et al. 2018). HHP processing for microbial control can pursue not only reducing the risk of wine spoilage (Mok et al. 2006; Tomašević et al. 2020) but also facilitating the implementation of new biotechnologies for wine fermentation (Bañuelos et al. 2016).

All the studies consulted in this review are schematically compiled in Table 2 that summarizes the observed results. As Table 2 provides exhaustive information on the sample characteristics, initial microbial load, and treatment conditions, this information is not always fully detailed throughout the text.

**TABLE 2:** Studies used in this review focused on high hydrostatic pressure (HHP) processing for microbial control of grapes, musts, or wines.

Substrate									HHP treatment					
							Microbial load				
Type		Sugar content (g L^−1^)	Alcohol content (%, v/v)	pH	Total SO_2_ (mg L^−1^)	Free SO_2_ (mg L^−1^)	Type	Initial concentration (cfu mL^−1^)	Pressure (MPa)	Holding time	Temperature (°C)	Storage after HHP treatment	Observed results	References
Grape	Whole Tempranillo grapes	n.i.	—	n.i.	n.i.	n.i.	Yeasts TAB LAB (naturally present in the grapes)	10^4^ ≈10^2^ ≈10^2^	200 400 550	10 min	≤30°C during processing Equipment thermostatized at 20°C	NO	Pressurization at 200 MPa reduced yeast population tenfold, while no yeasts were detected after treatments at 400–550 MPa Pressure treatments at 200–550 MPa reduce bacterial populations by ≈1 log cfu mL^−1^	Morata et al. (2015)
	Whole Tempranillo grapes	n.i.	—	n.i.	n.i.	n.i.	*Saccharomyces cerevisiae* *Schizosaccharomyces pombe* *Torulaspora delbrueckii* *Metschnikowia pulcherima* *Lachancea thermotolerans* (naturally present in the grapes)	≈10^2^ ≈10^2^ ≈10^2^ ≈10^2^ ≈10^2^	400	10 min	Equipment thermostatized at 20°C	NO	After pressurization at 400 MPa, wild yeast levels were from undetectable to lower than 10 cfu mL^−1^	Bañuelos et al. (2016)
	Pinot noir crushed grapes	n.i.	—	n.i.	n.i.	n.i.	Inoculated *S. cerevisiae* *Brettanomyces bruxellensis* *Kluyveromyces thermotolerans* *Lentilactobacillus hilgardii* *Oenococcus oeni* *Acetobacter aceti*	> 10^5^	551	10 min	n.i.	NO	After pressurization at 551 MPa, no viable cells were detected	Takush and Osborne (2011)
Must	Barbera grape must	177.2	—	3.0 3.5 3.0 3.0 3.5 3.5	200 200 0 80 0 80	n.i.	Inoculated cocktail of vegetative cells and sporified cultures of 13 yeasts, 3 bacteria, and 1 mold	4.5 × 10^6^ 3.18 × 10^6^ 14.63 × 10^6^ 4.62 × 10^6^ 24.20 × 10^6^ 3.18 × 10^6^	300, 400 300, 400 600 600 600 600	2 min 2 min 2 min 2 min 2 min 2 min	20°C before pressurizing	NO	Pressure treatments at 300–400 MPa did not avoid microbial growth, even in must added with 200 mg L^−1^ SO_2_ Pressurization at 600 MPa inactivated vegetative yeast cells in musts both with and without 80 mg L^−1^ SO_2_ added Spores of *S. pombe* resisted up to 700 MPa for 2 min	Delfini et al. (1995)
	Heat‐sterilized grape juice	n.i.	—	n.i.	n.i.	n.i.	Inoculated *S. cerevisiae* 4 strains (F, G, P, and B)	≈10^6^	180–300	1–6 min	RT before pressurizing 4°C (Equipment thermostatized)	NO	*S. cerevisiae* baroresistance depends on pressure applied, temperature, holding time, and yeast strain HHP treatments at 300 MPa for 5 min completely inactivated strains B and F. Pressurization at 4°C produced higher yeast inactivation than at RT	Tonello et al. (1998)
Wine	Sauternes white wine in the process of fermentation	70	12.3	3.9	n.i	n.i	Microflora naturally present during fermentation	7.5 × 10^6^ (yeasts) 2 × 10^6^ (yeasts)	200–400 300	5–20 min 1–15 min	RT before pressurizing	NO	No viable yeasts after pressure treatments at pressures of 300 MPa and higher for 10 min or longer	Lonvaud‐Funel et al. (1994)
	Moscato wines	50.5 50.5 50.5 0.0 0.0 0.0 0.0	8.22 8.22 8.22 11.35 11.35 15.19 15.19	3.1 3.1 3.1 3.1 3.1 3.1 3.1	64 64 167 102 192 102 192	n.i.	Inoculated cocktail of vegetative cells of 13 yeasts, 3 bacteria, and 1 mold	5.44 × 10^6^ 7 × 10^6^ 1.6 × 10^6^ 5.90 × 10^6^ 0.36 × 10^6^ 1.36 × 10^6^ 0.05 × 10^6^	300–500 600 600 600 600 600 600	2 and 4 min 3 min 3 min 3 min 3 min 3 min 3 min	20°C before pressurizing	NO	Lower microbial baroresistance in wine compared to that observed in must Pressurization at 400 MPa for 2 min produced complete microbial inactivation in sweet wine Pressurization at 600 MPa for 3 min produced complete microbial inactivation, whichever the sugar, SO_2_, or alcohol content of the wine	Delfini et al. (1995)
	Wine obtained by fermenting heat‐sterilized grape juice inoculated with commercial strains of *S. cerevisiae* for either 9 (D9) or 18 days (D18)	1.5 (D9) 0.9 (D18) 8–98 (added)	8.9 (D9) 9.2 (D18) 8.6, 10.8, 13, and 15 (added)	n.i.	n.i.	n.i.	*S. cerevisiae* naturally present after fermentation	n.i.	180–300	1–6 min	RT before pressurizing	NO	Lower *S. cerevisiae* baroresistance in D18 wines compared to D9 wines Alcohol contents greater than 13% reduced yeast baroresistance Sugar content, up to 98 g L^−1^, did not affect yeast inactivation	Tonello et al. (1998)
	Wine from Campbell Early grapes fermented at 25°C with *S. cerevisiae* KCCM12224 for 2 weeks and aged at 15°C for 14 weeks	0.851%	9.0	3.3	n.i.	n. i.	Total aerobes Yeasts LAB (naturally present in the wine)	4.15 × 10^5^ 2.87 × 10^5^ 2.86 × 10^5^	100, 150, 200, 250, 300, and 350	5, 10, 20, and 30 min	25°C before pressurizing	NO	The higher the pressure and the longer the holding time, the greater the inactivation attained Reducing wine microflora under the detection limits required 30 min at 300 MPa or 10 min at 350 MPa	Mok et al. (2006)
	DO Mancha red wine	n.i.	13	3.2 3.2 3.6 3.6	35	11	Inoculated *B. bruxellensis*	10^4^ 10^6^ 10^4^ 10^6^	100	24 h	25°C	NO	Complete inactivation and no formation of 4‐ethylphenol, whichever the wine pH (3.2 or 3.6) and the initial microbial load (10^4^ or 10^6^ cfu mL^−1^)	Morata et al. (2012)
	Synthetic wine	—	10 10 12 12 14 14	3.0 4.0 3.0 4.0 3.0 4.0	—	—	Inoculated *B. bruxellensis* 2 strains	≈10^6^	100, 200, and 300	1, 3, 5, 6, and 7 min	RT before pressurizing ≤35°C during processing	7 days at RT	Inactivation depended on pressure applied and holding time, wine characteristics (pH and ethanol content), and the yeast strain 300 MPa for 1 min produces complete inactivation, whichever the wine characteristics and yeast strain and no growth was detected after 7‐day storage High pH (4) combined with high ethanol content (14%) significantly reduced yeast baroresistance: only 100 MPa for 1 min produced complete inactivation, whichever the strain and no growth was detected after 7‐day storage	González‐Arenzana et al. (2016)
	Filter‐sterilized Cabernet Sauvignon wine	n.i.	13.4	3.5	n.i.	n. i.	Inoculated *B. bruxellensis* 3 strains (AWRI 1499, AWRI 1608, and AWRI 1613)	10^7^–10^8^	100 150 200 400 600	0–600 s 0–600 s 0–180 s 5 s 5 s	≤36°C during processing	NO	Pressure, time, and yeast strain had a significant effect on HHP inactivation Increasing pressure and holding time increased yeast inactivation The most and least resistant strains to HHP inactivation were AWRI 1499 and AWRI 1608, respectively 400 MPa and 600 MPa for 5 s produce complete inactivation of strain AWRI 1499 (> 6 log reduction)	van Wyk and Silva (2017a)
	Filter‐sterilized wines:	n.i.												
	Cabernet Sauvignon Syrah Pinot Noir Preservative‐free Cabernet Merlot Rosé Dolcetto Syrah Chardonnay		13.5 12.5 13.0 13.7 12.5 10.5, 12, 14 13.0	3, 3.5, 4 3.70 3.43 3.56 3.45 3.41 3.60	73 45 46 < 10 116 95 92	30 14 17 < 3 20 30 25	Inoculated *B. bruxellensis*, strain AWRI 1499	10^7^–10^8^	200	0–180 s	RT before pressurizing ≤24°C during processing	7 days (only in some samples)	Wine variety had a great effect on *B. bruxellensis* baroresistance Alcohol content above 12% increased HHP susceptibility of *B. bruxellensis* (Dolcetto Syrah wine) At similar alcohol content, increasing SO_2_ seems to reduce *B. bruxellensis* baroresistance pH, between 3 and 4, had little or no effect on HHP susceptibility of *B. bruxellensis* (Cabernet Sauvignon wine)	van Wyk and Silva (2017b)
	Filter‐sterilized SO_2_‐free Cabernet Merlot Australian red wine	n.i.	13.7	n. i.	< 10	< 3	Inoculated *B. bruxellensis*, strain AWRI 1499	2.3 × 10^5^–5.9 × 10^5^	400	5 s	RT before pressurizing ≤40°C during processing	1 year at 25°C	Complete inactivation (> 5 log reduction) after HHP treatment at 400 MPa for 5 s No recovery after 1‐year storage No 4‐EG or 4‐EP content after 1‐year storage	van Wyk et al. (2018)
	Filter‐sterilized Cabernet Sauvignon dry red wine	3.7	13.0	3.5	48	12	Inoculated *B. bruxellensis*, strain CBS 2499	3.2 × 10^5^	100 and 200	1, 3, 5, 15, and 25 min	n.i.	30, 60, and 90 days in sterile bottles at 20°C ± 2°C	Treatments at 100 MPa, even for 25 min, hardly affected ‘Brett’ viability “Brett” reached a VBNC state after treatments at 200 MPa for 15 and 25 min During storage, “Brett” culturability completely recovered	Tomašević et al. (2020)
	Filter‐sterilized sweet white wine Graševina	53.7	11.7	3.3	80	24	Inoculated *S. cerevisiae*, strain DSM 70468	2.5 × 10^4^	100 and 200	1, 3, 5, 15, and 25 min	n.i.	30, 60, and 90 days in sterile bottles at 20°C ± 2°C	Treatments at 100 MPa, even for 25 min, did not affect *S. cerevisiae* viability Treatments at 200 MPa, for 15–25 min, produced 1.5 log reduction and sublethal damage During storage, no culturable cells were detected in wines treated at 200 MPa for 15–25 min	

Abbreviations: “Brett,” *Brettanomyces bruxellensis*; 4‐EG, 4‐ethylguaiacol; 4‐EP, 4‐ethylphenol; LAB, lactic acid bacteria; n.i., not indicated; RT, room temperature; TAB, total aerobic bacteria.

#### 3.1.1 Prevention of Microbial Spoilage

Microbial spoilage of wine can occur throughout the entire vinification process and result in significant economic losses. Contaminating yeasts, molds, and bacteria can come from a variety of sources, including grape skins and stems, insects, winery walls, or equipment in contact with the grapes, must, or wine. Their potential adverse effects include off‐flavors and ‐odors, color degradation, film‐like growths on the wine surface, and the formation of flocculants, silky threads, and granular or viscous deposits, thus resulting in significant wine degradation (du Toit and Pretorius 2000; Cosme et al. 2018; Van Wyk and Silva 2019). To prevent microbial spoilage, traditional practices in the wine industry cover a wide range of tasks, including grape quality control, proper cleaning and sanitation of winery equipment, control and management of must/wine temperature, pH, and oxygen exposure, physical removal of microorganisms through filtration, and use of chemical and/or biological additives, such as SO_2_ or antimicrobial peptides, among others (du Toit et al. 2005). However, all these practices have limitations and/or drawbacks that encourage the search for new technologies capable of guaranteeing microbial stability. In this sense, HHP processing has proven to be particularly effective.

**FIGURE 3:**
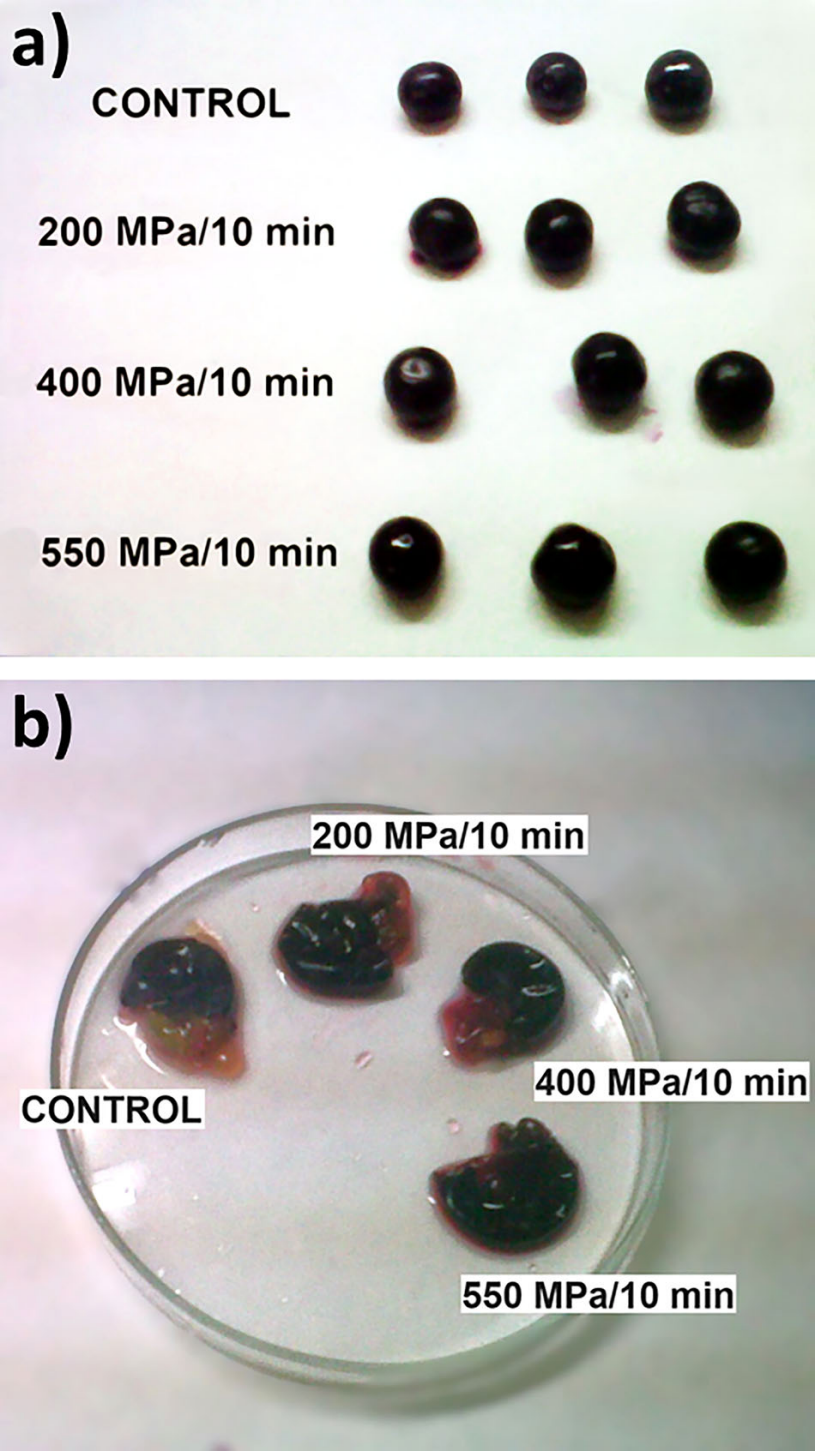
(a) Effect of HHP treatments, either at 200, 400, or at 550 MPa for 10 min, on grape shape and integrity; (b) grapes crushed before (control) and after HHP treatments either at 200, 400, or at 550 MPa for 10 min. Note differences between the pulp and seed color of control and pressurized grapes: not pigmented pulp and seed surface became stained after pressure processing.

**Grapes** can be pressurized, either before (Bañuelos et al. 2016; Morata et al. 2015) or after crushing (Takush and Osborne 2011), to reduce their wild microbiota, thus facilitating a more hygienic winemaking process. When applied before crushing, HHP does not produce berry breakage (Figure 3a), and, thus, grape shape and integrity remain intact after the treatment (Morata et al. 2015). Microbial inactivation achieved in the grapes after pressure processing depends on the pressure level applied and the holding time but also on the specific microbiota present in the product. Obviously, the higher the pressure and the longer the treatment, the larger the microbial inactivation attained. In this sense, Morata et al. (2015) found that pressurization of Tempranillo grapes, at 200 MPa and 20°C for 10 min, only reduced the initial wild yeast population from 10^4^ to 10^3^ cfu mL^−1^ but after 10 min at 400 MPa, no viable yeasts were detected. Bacteria showed greater resistance, and, thus, initial counts of 10^2^ cfu mL^−1^ only reduced 10‐fold, even after pressure treatments at 550 MPa. Bacteria most commonly associated with grapes are lactic acid bacteria (LAB), that is, Gram‐positive bacteria that are, generally, much more resistant to HHP than yeasts. Consequently, they require higher pressures to achieve complete inactivation. However, it should be noted that pressure resistance can vary considerably between species and, even, between strains of the same species. Furthermore, the specific characteristics of the substrate can also play a significant role. Thus, unlike Morata et al. (2015), Takush and Osborne (2011) observed that a 10‐min exposure at 551 MPa was sufficient to reduce viable cell counts in crushed Pinot noir grapes, inoculated with a cocktail of yeasts, LAB, and acetic acid bacteria (initial viable cell counts ≈ 10^5^ cfu mL^−1^), to below the detection limits. All these findings reveal that grape pressurization can be an effective strategy to avoid the detrimental effects of undesirable microbiota, thus opening a way for reducing the amount of SO_2_ added during winemaking. This is particularly interesting in red vinification where grape skins remain present during fermentation and the high polyphenolic content reduces the need for external antioxidants (Morata et al. 2015).

**Must** pressurization is also effective in inactivating microorganisms (Delfini et al. 1995; Tonello et al. 1998), thus allowing fermentation to be conducted solely by the microbiota that is subsequently inoculated. This maximizes its fermentative performance and makes it possible to avoid the influence of undesirable yeasts and bacteria naturally present in the must. However, the presence of spore‐forming microorganisms and the high sugar content of the must (160–250 g L^−1^), which could impart a baroprotective effect on its wild microbiota (Goh et al. 2007), require either high pressure levels or long holding times for microbial stabilization. In this sense, Delfini et al. (1995) reported that pressure treatments, at 400 MPa for 2 min, were not sufficient to completely inactivate a mixture of microorganisms (13 wine yeasts, *Leuconostoc oenos*, *Lactobacillus* spp., *Acetobacter* spp., and *Botrytis cinerea*) that had been inoculated both as vegetative cells and spores (≈10^6^ cfu mL^−1^) in Barbera grape must, even after the addition of 200 mg L^−1^ SO_2_. Complete inactivation of vegetative cells in the must required 600 MPa for 2 min, whereas mature spores of *Schizosaccharomyces pombe* survived up to 700 MPa for 2 min. Must pressurization at low temperature could help to increase microbial inactivation, although it is important to note that it would significantly increase the cost of the treatment. In this sense, Tonello et al. (1998) observed higher yeast inactivation in musts inoculated with 10^6^ cfu mL^−1^ of *S. cerevisiae* and pressurized at 4°C than in those treated at room temperature (both at 180–300 MPa for 1–6 min).

Pressurization of **wine** after fermentation can also help in avoiding microbial spoilage during maturation, aging, and/or the subsequent storage. As observed in grapes and must, the higher the pressure and the longer the treatment, the larger the inactivation attained in the microorganisms present in the wine (Delfini et al. 1995; Lonvaud‐Funel et al. 1994; Mok et al. 2006; Tonello et al. 1998). Thus, for example, reducing the naturally present aerobes, yeasts, and LAB (≈4 × 10^5^, 3 × 10^5^, and 3 × 10^5^ cfu mL^−1^, respectively) under the detection limits in a low‐alcohol wine required 20 min at 300 MPa, but only 10 min at 350 MPa (Mok et al. 2006). Moreover, it is also interesting to note that, due to its particular physicochemical characteristics, wine reduces microbial baroresistance compared to that observed in must (Tonello et al. 1998). Thus, at 400 MPa, Delfini et al. (1995) found that holding times as short as 2 min were enough to produce complete microbial inactivation in Moscato wine inoculated with a mixture of vegetative cells of wine yeasts, bacteria, and molds (initial load: ≈5 × 10^6^ cfu mL^−1^).

However, it should be highlighted that, due to the low pH and relatively high alcohol content of wine, only a few spoilage yeasts, LAB, and acetic acid bacteria are actually able to survive after alcoholic fermentation and cause quality problems during aging and storage. Among them, the yeast *Brettanomyces/Dekkera bruxellensis* (anomorphic/teleomorphic forms of the same organism) is one of the most concern, especially in red wines (Oelofse et al. 2008; Wedral et al. 2010). By enzymatic transformation, this yeast is able to convert hydroxycinnamic acids (*p*‐coumaric, ferulic, and caffeic acids) into volatile phenols (4‐ethylphenol, 4‐ethylguaiacol, and 4‐ethylcathecol, respectively). These volatile phenols impart unpalatable off‐odors and ‐flavors to the contaminated wine that are described as “medicinal,” “animal,” “horse sweat,” “spicy,” or “barnyard‐like.” This renders the wine unfit for consumption, resulting in significant economic losses (Agnolucci et al. 2017; Malfeito‐Ferreira 2018; Suárez et al. 2007). In addition, *B. bruxellensis* is capable of producing biogenic amines, which can cause adverse physiological reactions such as headaches, gastric disorders, or palpitations in individuals with amine intolerance (Oelofse et al. 2008).

*B. bruxellensis* poses a significant risk to wine stability due to its ability to survive and grow in anaerobic conditions in media with low pH, high alcohol content, and low nutrient content (Agnolucci et al. 2017). Furthermore, under stress conditions, cells can enter in a viable but non‐culturable (VBNC) state, which is characterized by the inability of cells to divide on selective media. In this state, cells remain viable despite exhibiting low metabolic activity and when environmental conditions are favorable, their growth is reinitiated. Despite the proven efficacy of SO_2_ addition in preventing *Brettanomyces* contamination during winemaking, its adverse effects on consumers and its potential to induce a VBNC state in the yeast (Agnolucci et al. 2014; du Toit et al. 2005) have aroused interest in exploring alternative technologies for *Brettanomyces* inactivation.

Several studies in the literature reveal the effectiveness of HHP in inactivating *B. bruxellensis* in wine (González‐Arenzana et al. 2016; van Wyk et al. 2018; van Wyk and Silva 2017a, 2017b). As observed for other microorganisms, *B. bruxellensis* inactivation is pressure and time dependent. Pressure as low as 100 MPa can achieve complete inactivation, but only if it is maintained for enough time. In this sense, van Wyk and Silva (2017a) and Tomašević et al. (2020) observed <1 log reductions in Cabernet Sauvignon wine after HHP treatments at 100 MPa for 10 and 25 min, respectively. However, after 24 h at 100 MPa, Morata et al. (2012) reported complete inactivation and no 4‐ethylphenol formation in a DO Mancha red wine. Application of higher pressures significantly reduces time needed to inactivate *B. bruxellensis* in wine, and, thus, no culturable cells were detected after pressure treatments either at 200 MPa for 15 min (Tomašević et al. 2020), at 300 MPa for 1 min (González‐Arenzana et al. 2016), or at 400 MPa for 5 s (van Wyk et al. 2018; van Wyk and Silva 2017a). However, Tomašević et al. (2020) reported that pressure treatments at 200 MPa for up to 25 min resulted in non‐complete inactivation but in entering a VBNC state, and, thus, after 30 days of storage, *B. bruxellensis* cells recovered culturability completely. By contrast, no evidence was found of pressure‐induced VBNC state in *B. bruxellensis* cells treated at higher pressures, and, thus, neither culturability recovery (González‐Arenzana et al. 2016; van Wyk et al. 2018) nor formation of 4‐ethylguaiacol and 4‐ethylphenol were reported in wines pressurized at ≥300 MPa during storage (van Wyk et al. 2018).

*Brettanomyces* inactivation depends not only on the treatment conditions but also on the yeast strain and the wine characteristics (van Wyk and Silva 2017a, 2017b). Thus, several authors have found that, depending on the strain, baroresistance of this yeast can significantly differ (González‐Arenzana et al. 2016; van Wyk and Silva 2017a). For example, after 5 min at 150 MPa, van Wyk and Silva (2017a) detected 2.0, 3.1, and 4.2 log reductions in red wines inoculated with strains AWRI 1499, 1608, and 1613, respectively. On the other hand, these authors also observed large differences among the log reductions attained in different red, white, and rosé wines treated at 200 MPa for up to 3 min (van Wyk and Silva 2017b). Thus, complete *Brettanomyces* inactivation (≥7 log reductions) occurred in Chardonnay wine (13% alcohol, 3.6 pH, 92 mg L^−1^ total SO_2_, 25 mg L^−1^ free SO_2_) after 15 s at 200 MPa, whereas, after 3 min, only 3 log reductions were observed in Dolcetto Syrah wine (10.5% alcohol, 3.41 pH, 95 mg L^−1^ SO_2_, 25 mg L^−1^ free SO_2_). These results suggest that both SO_2_ and alcohol content in the wine affect *Brettanomyces* baroresistance. In this sense, several authors have found that wine alcohol contents above 12% v/v can significantly increase log reductions observed after pressurization not only in *B. bruxellensis* (González‐Arenzana et al. 2016; van Wyk and Silva 2017b) but also on other yeasts (Tonello et al. 1998). Other wine characteristics, such as pH, could also play a significant role on the baroresistance of *Brettanomyces*. Thus, for example, González‐Arenzana et al. (2016) found that pressurization at 100 MPa for 1 min was enough to completely inactivate two strains of *B. bruxellensis*, isolated from Rioja red wines, when inoculated in synthetic wine with 14% ethanol and pH = 4, but no inactivation effect was observed in the same synthetic wine at pH = 3, even after 7 min at 100 MPa. These results contrast with those reported by van Wyk and Silva (2017b) who did not find any effect of pH, between 3 and 4, on *Brettanomyces* inactivation attained in Cabernet Sauvignon wine (13.5% alcohol, 73 mg L^−1^ total SO_2_, 30 mg L^−1^ free SO_2_). Combined effects of pH, SO_2_, and alcohol content could explain differences observed in the inactivation of *B. bruxellensis* in different substrates.

Another yeast that can pose a problem for wine stability is *S. cerevi*siae. It is usually the most abundant microorganism in wine after alcoholic fermentation, although other yeasts, such as *Zygosaccharomyces bailii*, for example, may also be present and together produce undesirable re‐fermentations, especially in sweet wines and bottled wines with residual sugars (Tomašević et al. 2020). Sugar present in the wine does not seem to increase the baroresistance of *S. cerevisiae*, and, thus, Tonello et al. (1998) found no differences in pressure‐induced inactivation observed in wines fortified with sugar between 8 and 98 g L^−1^. Immediately after pressurization at 200 MPa for either 15 or 25 min, Tomašević et al. (2020) reported a 1.5 log reduction in *S. cerevisiae* population inoculated in sweet white wine, but later, after 30–90 days of storage, no cultivable cells were detected. This suggests that sublethal damage occurred in the yeast cells immediately after pressurization, allowing them to grow on selective medium, but during wine storage their increased sensitivity led to cell death.

All these results reveal that HHP processing can effectively guarantee microbial stability during vinification, and therefore, this technology has been proposed to preserve wine with reduced amounts of SO_2_ (Christofi et al. 2020; Santos et al. 2012; Silva and van Wyk 2021; van Wyk et al. 2018). Due to its antiseptic, antioxidant, and antioxidase functions, this additive is frequently used in different stages of the winemaking process, from crushing to bottling (Giacosa et al. 2019; Silva and van Wyk 2021). However, it can cause allergic reactions, headaches, asthma, and other adverse effects in consumers, and, for this reason, the maximum permitted SO_2_ content in wine is limited by law in major wine‐producing countries. For example, depending on the sugar content, the European Commission (2019) legislation limits total SO_2_ to 150–200 and 200–250 mg L^−1^ in red and white/rosé wines, respectively. These limitations, together with the growing demand for healthier, additive‐free, organic products, have aroused the interest of the wine industry in new strategies capable of minimizing the use of SO_2_ in wine production. In this sense, several studies reveal that the doses of SO_2_ required for wine protection and preservation can be reduced by combining the antimicrobial action of HHP and the antioxidant and antioxidasic effect of sulfur dioxide (Christofi et al. 2020). Complete replacement of SO_2_ use is not feasible because its antioxidant and antioxidasic effects are not provided by HHP processing. In fact, as explained further below, several studies reveal that HHP processing increases the rates of condensation and oxidation reactions during wine storage, and thus, HHP‐treated wines may be perceived as less aromatic, brownish, and more oxidized if insufficient doses of SO_2_ are added before storage (Christofi et al. 2020; Santos, Nunes, Rocha, et al. 2013; van Wyk et al. 2018).

#### 3.1.2 Use of New Biotechnologies for Wine Fermentation

It is common practice in the wine industry to use commercial starters of *S. cerevisiae* for must fermentation. This method avoids the risk of spoilage associated with spontaneous fermentation and ensures highly predictable and reproducible processes. These starters are generally selected for their fermentative power, suitable fermentative kinetics at different temperatures, low acetic acid production, and resistance to sulfur dioxide (Suárez‐Lepe and Morata 2012). However, as a side effect, they reduce the potential of vineyard yeasts to impart their specific fingerprint to the wine and, due to their limited number, give rise to wines with similar analytical and sensory characteristics (Comitini et al. 2017). This problem is currently aggravated by those associated with climate change (loss of freshness, flat sensory profile, or high ethanol content in wines, among others) that contribute to reduced wine quality. This is why, in recent years, interest has been aroused in new biotechnologies for wine fermentation (e.g., use of new *S. cerevisiae* strains, non*‐Saccharomyces* yeasts, yeast‐bacteria co‐inoculations) that make it possible to meet the growing demand for high‐quality, sensory‐complex wines with differential identity characteristics.

Yeast selection is, nowadays, focused not only on *S. cerevisiae* strains but also on non‐*Saccharomyces* species capable of improving the aroma, color, structure, and other sensory properties of wine. In this sense, specific yeast species can be used to enhance the fermentative formation of aromatic compounds, increase the synthesis of stable pyranoanthocyanin pigments, or improve the wine structure by releasing polysaccharides and forming polyalcohols (Morata and Suárez‐Lepe 2015). However, it is important to note that the use of non‐*Saccharomyces* yeasts for must fermentation can be seriously hampered by the presence of wild grape yeasts, which often exhibit superior fermentative power and/or faster fermentation rates. For this reason, HHP technology has been proposed to reduce the wild microflora in grapes, thus enhancing the potential for the use of non‐*Saccharomyces* yeasts in oenology. In this sense, Bañuelos et al. (2016) pressurized Tempranillo grapes (initial wild yeast load: 10^2^ cfu mL^−1^) at 400 MPa for 10 min and observed that wild yeast counts were reduced to below 10 cfu mL^−1^. Musts obtained from crushed grapes, pressurized or not, were then inoculated with different yeasts, either used individually as sole fermentative yeasts (*S. cerevisiae* and *S. pombe*) or used in sequential cultures with *S. cerevisiae* (*Torulaspora delbrueckii*, *Metschnikowia pulcherrima*, and *Lachancea thermotolerans*) for complete fermentation of sugars. HHP treatments at 400 MPa for 10 min limited the significant growth of wild yeast populations observed during fermentation of control musts, allowing better implantation and development of inoculated yeasts. This enhanced their metabolic expression and consequently affected the quality of wine. Thus, for example, Bañuelos et al. (2016) observed that HHP was particularly effective in enhancing the activity of inoculated *L. thermotolerans*, almost doubling the lactic acid produced in musts from grapes not treated with HHP. Grape pressurization also allowed to improve the wine aroma profile by increasing the concentrations of acetoin, 3‐ethoxy‐propanol, or 2‐phenylethyl alcohol as a consequence of the HHP‐enhanced activity of *S. pombe*, *T. delbrueckii*, or *M. pulcherrima*, respectively.

All these results clearly demonstrate the enormous potential of HHP in the development of new biotechnologies for wine fermentation. HHP can drastically reduce wild grape microflora, thus promoting the successful inoculation and implantation of specific yeast or bacterial starters especially designed to improve alcoholic or malolactic fermentation. Furthermore, by avoiding the interference of wild microbiota, better expression of their enzymatic activities and greater production of desired metabolites can be achieved, thus improving wine quality.

### 3.2 HHP Processing to Enhance Extraction

HHP processing has also been envisaged as a promising extraction methodology to recover bioactive compounds from vegetal substrates (Khan et al. 2019; Martín and Asuero 2021). As previously commented, the HHP‐induced rupture of ionic bonds and hydrophobic interactions can result in conformational changes at the molecular level, thus enhancing permeability of cellular membranes by making them less selective. In addition, in vegetal tissues, the air trapped between cells significantly reduces its volume during compression, causing air gaps to be partially filled with fluid. When pressure is released, the trapped air expands instantaneously, damaging the integrity of the cell walls. Small pores or fissures in the cell walls, together with the pressure‐enhanced permeability of the cell membranes, facilitate the extraction of metabolites from plant cells. For these reasons, HHP technology has been successfully employed to improve the extraction of bioactive compounds from plant matrices such as pomegranate peels (Alexandre et al. 2017), stinging nettle leaves (Moreira et al. 2020), or papaya seeds (Briones‐Labarca et al. 2015), among others. As solid–liquid extraction phenomena are essential for wine elaboration, a number of researchers have evaluated the potential of HHP to improve mass‐transfer processes in critical steps of the winemaking process such as must maceration or aging (Morata et al. 2023; Valdés et al. 2021; Voce et al. 2024).

#### 3.2.1 Must Maceration

During maceration, essential molecules that define the organoleptic characteristics of wine, including pigments, tannins, and aromatic compounds, migrate from the grape skins to the must. This step is particularly important in red vinification as anthocyanins, flavonoids responsible for wine color, are located at the vacuoles of the epidermal skin cells in most of grape varieties (only a few varieties also have anthocyanins in the pulp). Phenolic compounds extracted during maceration not only affect wine color, taste, or structure but also its antioxidant capacity and barrel‐aging capacity, among others (Morata et al. 2019; Nel 2018). Therefore, wine quality strongly depends on the efficiency of mass‐transfer processes during the maceration step.

Several studies in the literature prove that HHP, applied on entire grapes, can significantly improve solid–liquid extraction phenomena during maceration. By damaging the integrity of cell walls and increasing the permeability of tonoplasts and cell membranes, HHP can eliminate physical barriers against diffusion in the epidermal cells, thus enhancing the migration of phenolic and aromatic compounds from the grape skin toward the pulp. In this sense, Morata et al. (2015) observed that, just after pressurization (either at 200, 400, or 550 MPa for 10 min), the surface of Tempranillo grapes was brighter, whereas both the not pigmented pulp and the seed surface became stained (Figure 3b). Accordingly, the total anthocyanin content in HHP‐treated grapes almost doubled, after crushing and before maceration, that observed in control grapes. Moreover, no effect of pressure level on the anthocyanin content was detected, thus suggesting that the pressure‐induced mechanical damage to the skin cell walls may have reached its maximum at 200 MPa. During maceration/fermentation, compound extraction from the grape skins continued, and at the end of fermentation, all the wines obtained from HHP‐treated grapes had, whichever the pressure, ≈65% higher total phenolic (TP) index, 68% higher *p*‐coumarylated, and ≈20% higher total anthocyanin content than those measured in the control wine.

Even though pressure level applied on the grapes seems not to affect the extraction of skin compounds, holding time significantly does. Thus, Takush and Osborne (2011) observed that the longer the pressurization of crushed Pinor noir grapes at 551 MPa, the greater the amount of phenolic compounds extracted during maceration. Thus, they reported increments of 20% and 72.4% in wines obtained from grapes pressurized for 5 and 10 min, respectively.

Pressure‐enhanced extraction of phenolic compounds during maceration could not only shorten this step of the winemaking process, but it can also be useful when working with grapes poor in polyphenols. Thus, by optimizing the extraction process, the need to blend with a grape variety richer in phenolic compounds or the use of pectolytic enzymes can be eliminated (Morata et al. 2017).

#### 3.2.2 Wine Aging

Pressure‐enhanced solid–liquid extraction can also be exploited during wine aging. This process aims at improving the sensory profile and mouthfeel of wines by allowing redox, esterification, condensation, polymerization, and other reactions to occur during a certain storage period. During aging, wine organoleptic characteristics evolve and are harmonized to achieve the desired balance of aromas and flavor. Traditionally, aging has been carried out by storing wines in wooden barrels for several months or years. Despite its significant and recognized benefits on wine quality, this technique is very time‐consuming and expensive, mainly due to the high cost and space requirements of barrels. Additionally, in some ways, it is also risky because some microorganisms such as *Brettanomyces* can proliferate during aging. For these reasons, there is an increasing interest in alternative technologies able to shorten the aging process, thus improving the production capacity and economic benefits of wineries (Krüger et al. 2022; Ma et al. 2022).

One of these alternatives is **wine aging with oak wood fragments** (chips, cubes, shavings, granules, blocks, or boards), practice authorized in Europe since the early 2000s (European Commission 2006, 2019). Addition of wooden pieces into the wine container accelerates the release of wood‐related compounds (ellagitannins, furfural compounds, guaiacol, eugenol, among others) into the wine and allows reducing production costs associated to barrels. Several studies in the literature have demonstrated that wine aging with wood chips can be carried out under pressure, and this further speeds up the extraction process (Tao et al. 2016; Valdés et al. 2021). Thus, for example, Valdés et al. (2021) observed that a 5‐min maceration of holm oak chips (5 g L^−1^) in Cayetana white wine at 400 MPa increased the initial contents of catechins, flavonoids, hydroxycinnamic compounds, and total polyphenolics by 48.9%, 27.4%, 8.6%, and 19.2%, respectively. By contrast, conventional maceration of this wine with holm oak chips for 45 days did not affect its polyphenolic composition. Obviously, the characteristics and composition of wine exert a considerable influence on pressure‐induced extraction. In this sense, the same authors observed that catechin, tannin, and total polyphenol contents in Tempranillo red wine increased after pressurization with holm oak chips at 400 MPa for 5 min, but in a much lesser extent than in white wine. The lower phenolic content in white wine is probably the main cause of the more intense transference observed from the chips, and, probably, larger pressure, longer time, and/or higher wood chips doses would be needed to obtain remarkable extraction of phenolic compounds in red wines (Valdés et al. 2021).

Data in the literature reveal that pressure level and holding time can significantly affect performance of HHP‐assisted phenolics extraction from wood chips to wine. In general, higher pressures accelerate extraction, and, thus, Tao et al. (2016) observed higher TPs, tartaric esters, and flavonol contents in young red wines treated with oak chips at 650 MPa for 15 min than in those pressurized at 250 or 450 MPa. Extending pressure holding time over 15 min did not increase phenolics extraction at 650 MPa, but a progressive increase was observed in wines treated at 250 and 450 MPa. Thus, after 45 min, the contents of tartaric esters and flavonols detected in these wines were higher than in those treated at 650 MPa. This is probably due to the fact that, at this pressure, chemical oxidation of polyphenols, induced by pressure‐generated radicals, occurred. Particularly interesting is the impact of the holding time on the anthocyanin content and composition. During maceration, wine anthocyanins can firstly be adsorbed by the oak chips, resulting in a reduction in their concentration. Subsequently, they can be desorbed and react with other oak components, thereby modifying their profile. Thus, after 5 min of maceration with oak chips at 250 MPa, Tao et al. (2016) observed a significant decrease in polymeric and total anthocyanins of red wine, whereas, after 45 min, significant increases in monomeric, polymeric, and total anthocyanins were detected.

The effects of wine maceration with oak chips under pressure are evident not only immediately after the treatment but also during bottle storage. Thus, after 6 months of storage, Valdés et al. (2021) measured higher contents of flavonoids, hydroxycinnamic acids, and total phenols in white wines macerated with holm oak chips, either conventionally for 45 days or at 400 MPa for 5 min, than in control non‐macerated wines. Polyphenolic contents were slightly higher in conventionally macerated wines, but differences with pressurized wines were in no case higher than 18%. These results clearly show that wine aging with holm oak chips under pressure for only some minutes yields similar results than those obtained at atmospheric conditions for 45 days, thus proving that HHP can significantly accelerate wine aging with wood chips.

**Aging on lees** (AOL) is another process that can take advantage of HHP. Traditionally, this winemaking practice consisted of aging wines onto the fermentation lees, that is, the residue formed at the bottom of the fermentation tank, mainly composed of microorganisms (mostly dead yeasts), tartaric acid, inorganic salts, and other organic residues (Comuzzo et al. 2022). During aging, yeasts undergo autolysis, and different substances such as cell wall polysaccharides and mannoproteins, antioxidant compounds, or aroma precursors, among others, are released. These substances can then interact with various components of the wine, thus contributing to its sensory evolution. However, conventional AOL is a slow process that usually requires, at least, 7–9 months before its effects can be appreciated in the sensory profile of wine. Consequently, it is prone to microbial contaminations due to the abundant presence of nutrients and viable microorganisms in the lees (Comuzzo et al. 2022; Morata et al. 2019). To avoid these inconveniences, the use of externally produced pure yeast biomasses, with improved performance for AOL, is an alternative to lees generated during wine fermentation. This option prevents for collateral contaminants and by selecting yeast species (even non‐*Saccharomyces* yeasts) and controlling doses, it guarantees fast autolysis and positive sensory impact. Moreover, the aging process can be further accelerated by applying chemical (enzyme addition) or physical autolysis induction treatments (ultrasound, pulse electric fields, high‐pressure homogenization, among others) to these biomasses, which provoke the breakage of the yeasts and the fragmentation of the cell walls (Comuzzo et al. 2015; del Fresno et al. 2019; Martínez et al. 2016; Morata et al. 2019). In this sense, HHP processing of yeast biomasses, even at pressures as low as 100 MPa, has been envisaged as a practice with great potential for accelerating wine AOL (Morata et al. 2019). However, recent studies reveal the results are, in some way, contradictory. Thus, Blanco‐Huerta et al. (2024) found that treating *S. cerevisiae* cells in a model wine system at 400–600 MPa for 3–10 min did not increase the nucleic acids, proteins, and total polysaccharides released during simulated AOL for 42 days, but rather the opposite. Thus, untreated samples presented higher concentrations of nucleic acids, proteins, and total polysaccharides throughout the entire aging process than those HHP‐treated, regardless the pressure level and holding time applied. These results agreed well with the environmental scanning electron microscopy images obtained after 42 days of simulated aging. These images showed yeast cells better preserved, less disordered structures, and less irregular fragments in pressure treated samples than in untreated controls. The authors suggested that HHP treatments could induce denaturation of enzymes responsible for cell autolysis, thus justifying these results. By contrast, Voce et al. (2024) observed that adding lees, obtained either by single (*S. cerevisiae*) or sequential *(Hanseniaspora uvarum* followed by *S. cerevisiae*) fermentation and treated at 400 MPa for 8 min, during AOL significantly improved the color evolution and volatile profile of white wine. Thus, these authors noted that the color of wines aged for 3 months on HHP processed lees differed from that of wines aged on either untreated or enzymatically treated lees, exhibiting the lowest yellow hue values. HHP processing enhanced the release of glutathione (GSH) in lees obtained from sequential fermentation, thus imparting a protective effect against wine oxidation. Moreover, it promoted the highest release of polysaccharides into the wine and magnified floral and fruity odor attributes and aftertaste. Inconsistent results reported by Blanco‐Huerta et al. (2024) and Voce et al. (2024) could be due to differences in the medium in which yeast cells were pressure treated, the aging time, or the presence of *H. uvarum* in the lees, and they highlight the necessity for further investigation on this topic.

#### 3.2.3 Exploitation of Winery By‐Products

Even though not directly involved in the winemaking process, HHP can also be exploited for extraction of bioactive substances from winery by‐products such as grape pomace. Thus, for example, Corrales, Toepfl et al. (2008) pressurized grape skins in a mixture of ethanol and water (50:50, v/v) at 600 MPa and 70°C for 1 h and observed that the TP content in the extract increased almost by 50% compared to that measured in control extracts obtained at atmospheric pressure.

It is important to note that processing conditions can affect the extraction yield considerably (Corrales et al. 2009; Putnik et al. 2018). In this sense, Corrales et al. (2009) observed that increasing temperature (20–70°C), extraction time (30–90 min), and solvent concentration (20%–80%) significantly enhanced diffusion of phenolic compounds in red grape skins, thus increasing the antioxidant capacity measured in the extracts obtained. Effects of pressure intensity during extraction (200–600 MPa) were much less marked, probably because pressure‐induced damage on cell walls and membranes already occurred at the lower pressure tested. However, the detailed analysis of the extracts revealed that pressure level had a selective effect on anthocyanin extraction based on the linked glucose moieties. Thus, the extraction of anthocyanin monoglucosides was enhanced at 200 MPa, whereas, at 600 MPa, maximal extraction of acylated anthocyanin monoglucosides occurred.

### 3.3 HHP Processing to Induce Chemical Changes

Wine composition is complex, and it is constantly changing throughout the winemaking process. During conventional aging, intricate chemical reactions occur between wine components, mainly affecting phenolic compounds’ composition and abundance. In this phase, phenolic compounds can precipitate, be oxidized, or participate in different condensation, polymerization, and co‐pigmentation reactions, thus improving organoleptic properties such as wine color, aroma, or flavor (Carpena et al. 2020). However, wine aging is, as previously commented, a lengthy and expensive process, and, for this reason, many investigations are being carried out to seek for new technologies capable of accelerating it, while ensuring wine quality (Ma et al. 2022).

In the last decade, a number of researchers have analyzed how pressure processing affects chemical reactions and interactions between wine components to evaluate the potential of HHP technology to artificially shorten the aging time and improve wine quality (Lukić et al. 2020; Santos et al. 2016; Tao et al. 2016). As phenolic compounds determine organoleptic characteristics and quality of wine, most studies focus on changes caused by pressure in these compounds. In general, the studies conclude that pressure processing reduces the TPs content and antioxidant activity of wines. When wines are treated under severe pressure conditions (≥600 MPa for > 15 min), these changes are observed immediately after the treatment, whereas, when pressure treatments are less intense, these changes are only detected after several months of wine storage. Thus, Lukić et al. (2020) observed a significant decrease of 4.8% in TPs of red Cabernet Sauvignon wine just after pressurization at 600 MPa for 25 min, whereas Tao et al. (2012) noted a similar decrease in Nero D'avola‐Syrah red wine after processing at 650 MPa for 120 min. Chemical oxidation of polyphenols, induced by high‐reactive radicals generated during severe HHP treatments, has been proposed as the underlying mechanism responsible for these results (Tao et al. 2012). By contrast, no effect on the TP content of both red and white wines was reported immediately after pressure processing at less severe conditions (Briones‐Labarca et al. 2017; Christofi et al. 2021; Santos et al. 2016; Santos, Nunes, Rocha, et al. 2013; van Wyk et al. 2021), and, at least, 6 months of storage were generally required to observe significant changes. For example, Santos, Nunes, Rocha et al. (2013) did not observe, just after treatment, significant changes in the TP content or the antioxidant activity of red wines pressurized, either at 425 or at 500 MPa, for 5 min. However, after 9 months of bottle storage, TP content in pressurized wines was 9% lower than in untreated ones. Christofi et al. (2019) reported similar results in red wine pressurized at 350 MPa for 10 min. Thus, just after the pressure treatment, no effect on TP content or antioxidant activity was observed, but after 12 months of storage, TP content and antioxidant activity in pressurized wines were 16% and 8% lower than in untreated ones, respectively.

When specific phenolic families are studied separately, data in the literature reveal that wine pressurization, at room temperature, can significantly affect phenolic acids, tartaric esters, flavan‐3‐ols, proanthocyanidins, anthocyanins, and flavonols. Moreover, depending on the pressure level and holding time, changes can be detected, as previously mentioned, either immediately or only after several months of storage (Lukić et al. 2020; Tao et al. 2013). For example, Tao et al. (2013) observed significant decreases of 10.2%, 9.8%, 5.0%, and 10.3% in tartaric esters, tannins, total anthocyanins, and flavonols contents, respectively, just after pressurizing red wine at 650 MPa for 2 h. By contrast, Santos et al. (2016) hardly noted changes in the phenolic acids, flavanols, monomeric anthocyanins, and flavonols contents of red wine treated at 600 MPa for 20 min, and only after 5 months of bottle storage were significant differences between untreated and pressurized wines detected.

However, it is important to note that pressure effects depend not only on processing conditions but also on the specific characteristics and composition of the treated wine. The complexity of the wine matrix offers a plethora of interactions able to occur among the multiple wine constituents, and, in consequence, general conclusions are frequently difficult to draw, and results reported by different authors in different wines often differ. In general, changes observed in pressurized wines, either immediately after processing or during storage, are similar to those occurring during conventional aging. However, as pressure can enhance some degradation and stabilization reactions, these changes occur more rapidly (Lukić et al. 2020; Santos et al. 2016). For example, after 5 months of bottle storage, Santos et al. (2016) noted reductions of 10.6%, 16.4%, 8.1%, and 41.3% in the phenolic acids, flavonols, mono‐ and oligomeric flavan‐3‐ols, and monomeric anthocyanin content of untreated red wine, whereas, in wine pressurized at 500 MPa for 5 min, these reductions were 17.9%, 28.2%, 12.8%, and 49.6%, respectively. The accelerated decrease of phenolic acids and flavonols during storage of pressurized wines suggests that HHP processing could enhance either the oxidation of these compounds or some reactions in which these compounds are involved, such as polymerization, esterification, or condensation reactions. Likewise, high pressure could accentuate oxidation of flavan‐3‐ols but also boost condensation reactions between flavan‐3‐ols and anthocyanins and polymerization reactions. In this sense, several researchers have observed that the mean degree of polymerization in condensed tannins of red wine increases significantly after pressurization, especially at pressures greater than 400 MPa (Chen et al. 2012; Santos et al. 2016). On the other hand, the more rapid decrease of monomeric anthocyanins in pressurized wines suggests that high pressure could enhance degradation reactions and/or multiple condensation reactions in which they can participate. Moreover, several authors have shown that the content of copigmented anthocyanins generally increases in pressurized wines (Tao et al. 2012, 2013). Copigmentation is an electrostatic interaction that happens in wines between anthocyanins and some molecules, called copigments (phenols, flavonoids, cations, and alkaloids), without the formation of chemical bonds. It promotes a hyperchromic and bathochromic shift in the maximum wavelength of absorption, giving red bluish colors that are much appreciated by the consumer and therefore have a positive impact in wine color quality (Escribano and Santos Buelga 2012). In this sense, He et al. (2018) observed that pressurization of model solutions, at 300 MPa for 2 min, promoted rapid copigmentation between anthocyanins of *Vitis amurensis* Rupr and organic acids. Furthermore, some studies indicate that high pressure could also enhance cycloaddition reactions between anthocyanins and pyruvic acid, thus producing pyranoanthocyanins, highly stable adducts with interesting properties for color improvement in red wines (Corrales, Butz, et al. 2008; Liu et al. 2018). Nevertheless, this process necessitates prolonged pressure exposure at elevated temperatures. Thus, for example, Corrales, Butz et al. (2008) subjected model solutions of cyanidin‐3‐*O*‐glucoside (Cy3gl) and pyruvate to 600 MPa and 70°C for over 30 min and noted that approximately 25% of Cy3gl was degraded and a vitisin A‐type derivative was formed. These authors also observed that pressurizing red wine at 600 MPa and 70°C for 1 h resulted in a slight, although not statistically significant, reduction in monomeric anthocyanin content. However, shorter pressure holding times, specifically 10 min, did not produce any discernible effect. Moreover, Morata et al. (2012) also noted no increase in vitisin A content in red wine treated at 100 MPa for 24 h. These results were later corroborated by Liu et al. (2018) who analyzed the effect of HHP processing, at 350–550 MPa and 25–55°C for 30–90 min, on the formation of vitisin A in young wine supplemented with pyruvic acid. They concluded that HHP processing can promote vitisin A formation, but only when high pressures are combined with high temperatures and maintained for an extended period. However, these extreme conditions appear to be impractical for wine production, as they are likely to impact the organoleptic properties of the wine.

As occurs in untreated wines, chemical changes observed in pressurized wines during storage largely depend on the SO_2_ presence and concentration, and the higher this is, the smaller the changes observed. In this sense, Lukić et al. (2020) compared the evolution of main phenolic compounds in wines with different concentrations of free SO_2_, pressurized or not at 200 MPa for 5 min, during 12 months of storage. In red wines (either 10 or 25 mg L^−1^ of free SO_2_), they found that TPs, total anthocyanins, total tannins, monomeric anthocyanins, and flavanols decreased during storage, and this decrease was significantly more pronounced in pressurized wines, especially in those with lower SO_2_ content. In white wines (either 25 or 45 mg L^−1^ of free SO_2_), they observed similar results, and, thus, the decrease in TPs and flavanols during storage was significantly higher in pressurized wines with lower SO_2_ content. Accordingly, Christofi et al. (2020) also noted that, after 12 months of storage, red wine with no added SO_2_, pressurized at 350 MPa for 10 min, presented contents of flavanols and monomeric anthocyanins 71% and 86% lower than those measured in untreated wine. By contrast, in wines containing 100 mg L^−1^ of SO_2_, no differences between pressurized and untreated wines were detected after 12 months of bottle storage. All these results suggest that SO_2_ addition counteracts the acceleration that pressure treatments cause in multiple chemical reactions implying phenolic compounds during wine storage, especially in oxidation reactions.

Considering the current trend to reduce SO_2_ content in wines, some recent studies have raised the possibility of complementing HHP processing and lower SO_2_ doses with GSH, a natural antioxidant in grapes capable of limiting browning and typical off‐flavors during aging. Thus, Christofi et al. (2021) noted that HHP processing at 400 MPa for 5 min, together with doses of 10 mg L^−1^ of GSH combined with ≥80 mg L^−1^ of SO_2_ allowed reducing, by approximately 20%, the decrease observed in anthocyanin content of red wine after 12 months of storage. However, this combination was not effective at lower doses of SO_2_. In this sense, Lukić et al. (2020) also observed that the addition of 20 mg L^−1^ of GSH to wines with low SO_2_ content (10 or 25 mg L^−1^ in red or white wines, respectively) only slightly reduces chemical changes during storage.

High‐pressure processing affects not only the evolution of the phenolic profile during wine storage but also other compounds. Thus, for example, Santos, Nunes, Rocha et al. (2013) noted that, after 9 months of storage, the total content of free amino acids in white wines pressurized, either at 425 or at 500 MPa, for 5 min was 18% and 23% lower than that in untreated samples, respectively. Moreover, pressurized wines presented higher content of furans. Specifically, the 2‐furfural content in wines processed at 425 and 500 MPa was 10‐ and 5‐fold higher than that measured in control wine, respectively, and 5‐methylfurfural and 2‐acetyl‐5‐methylfuran were only detected in the pressurized wine samples. All these results suggest that HHP treatments could enhance the Maillard reaction, which involves condensation of reducing sugars with amino acids, during storage, thus accelerating the decrease of the amino acid content and the formation of volatile Maillard compounds such as 2‐furfural. This hypothesis is also supported by the more brownish color observed in pressurized wines as the Maillard reaction results in the formation of melanoidins that produce a distinctive browning and also bready, roasted, and caramel aromas that may be sensorially related to oxidation and aging (Charnock et al. 2022).

Moreover, as previously mentioned, HHP processing can also induce structural changes in proteins. Although covalent bonds remain generally unaltered, other bonds and chemical interactions, such as ionic bonds and hydrophobic interactions, which are responsible for the secondary, tertiary, and quaternary protein structure, can undergo significant modifications. This results in conformational changes and, consequently, in the alteration of the intrinsic properties and functionality of proteins. In this sense, Tabilo‐Munizaga et al. (2014) demonstrated that, depending on the intensity of the treatment, HHP processing can modify the structure and thermal stability of wine proteins, thus affecting wine stability. They observed that pressure treatments, at 450 MPa for 3–5 min, significantly modified the secondary structure of proteins in Sauvignon Blanc wine by decreasing α‐helix structures and increasing turn, intermolecular, and intramolecular β‐sheet structures. These pressure‐induced structural changes improved thermal stability of wine proteins and, consequently, delayed haze formation. Thus, the authors concluded that HHP processing could improve colloidal stability and clarity of wines.

Data in the literature also reveal that pressure treatments can affect the evolution of wine aroma during aging (Christofi et al. 2020; Lukić et al. 2020; Santos et al. 2015). In general, the volatile profile of white and red wines is not significantly altered just after HHP processing, but differences between untreated and pressurized wines usually increase as the storage time progresses (Briones‐Labarca et al. 2017; Santos et al. 2015). For example, Santos et al. (2015) noted that, after 2 months of bottle storage, the volatile profiles of SO_2_‐free white and red wines pressurized, either at 425 or at 500 MPa, for 5 min were quite similar to that of untreated wines, but, after 9 months, remarkable differences were detected. Pressurized wines presented higher concentrations of acetals, ketones, furans, and aldehydes than those measured in untreated wines. These differences suggest that HHP processing accelerated the Maillard reaction and the oxidation of alcohols and fatty acids, thus leading to wines with a volatile composition characteristic of faster aged/thermally treated wines.

## 4 Effects of HHP Processing on Organoleptic Characteristics of Wine

Organoleptic characteristics are doubtless one of the most important factors influencing consumer perception of wine quality. Sensorial attributes of wine result from the unique and non‐linear interaction of the numerous molecules present in the wine matrix, with phenolic compounds playing a particularly relevant role. As described in previous sections, either grapes, musts, or wines can be pressure treated, and, depending on the pressure level and holding time, HHP processing can significantly affect extraction phenomena, chemical reactions, and interactions among wine components during the winemaking process. For this reason, a number of researchers have evaluated the effects of HHP processing on the organoleptic characteristics of wine (Mok et al. 2006; Morata et al. 2015; Santos et al. 2013; Tao et al. 2013; Valdés et al. 2021).

**Wines obtained from pressurized grapes** generally have higher content in certain phenolic compounds than those elaborated from untreated grapes. This is due to the effects that pressure causes in the grapes at the cellular level (increased permeability of cell membranes and pores and fissures in the cell wall) that favor their extraction. Thus, for example, Takush and Osborne (2011) observed that the TP content in young wines obtained from Pinot noir grapes was almost the double when the grapes were pressurized at 551 MPa for 10 min. Obviously, this higher phenolic content can significantly affect sensory attributes of wine. In this sense, Morata et al. (2015) reported that color intensity of red Tempranillo wine increased by 26% when it was elaborated from grapes pressurized, either at 200, 400, or at 550 MPa for 10 min. By contrast, Takush et al. (2011) did not detect significant color differences between wines made with HHP‐treated or untreated grapes, probably because, unlike Morata et al. (2015), these researchers added 30 mg L^−1^ of SO_2_ after wine fermentation.

Grape pressurization before vinification can also have significant effects on the volatile profile of the resulting wine, consequently affecting its aroma. These effects seem to depend on the pressure level applied. Thus, Morata et al. (2015) detected a lower content of total volatiles in wines made with grapes treated at 400 and 550 MPa than in those elaborated from 200 MPa‐treated or untreated grapes. They also observed that grape pressurization did not affect the content of higher alcohols in wine, but the ester content was higher in wines obtained from grapes pressurized at 200 and 400 MPa.

Sensory analysis of wines obtained from pressurized and untreated grapes corroborates all these results. Thus, Morata et al. (2015) reported that, although no significant differences were detected in astringency, bitterness, or tannicity, panelists perceived stronger color intensity and more fruity and less herbaceous aromas in wines made with HHP‐treated grapes. This resulted in a better global perception of these wines. Takush et al. (2011) observed similar results, and, thus, they described more intense fruity aromas in wines elaborated from 551 MPa‐treated grapes than in those made from untreated grapes. Moreover, they did not notice any effect on astringency, probably because all the wines were relatively young.

Several authors have shown that **HHP processing of wine** can lead to significant changes in its organoleptic characteristics. As observed for pressure‐induced chemical changes (Section 3.3), changes in organoleptic properties are immediately noticeable only after severe pressure treatments, whereas modifications in sensory attributes induced by less intense treatments are detectable after several months of storage. Thus, a number of researchers have reported that pressure processing of red and white wines at 300–500 MPa for up to 15 min hardly affects instrumental color measurements (Briones‐Labarca et al. 2017; Christofi et al. 2020; Tomašević et al. 2020). By contrast, higher pressures or longer holding times can cause significant changes in wine color immediately after treatment (Lukić et al. 2020; Tao et al. 2013; van Wyk et al. 2021). For example, Lukić et al. (2020) compared the color before and after different pressure treatments in both red and in white wines and found color differences (ΔE*) greater than 3, that is, perceptible to the naked eye, in wines treated at 600 MPa, but not in those treated at 200 and 400 MPa for 5–25 min.

Sensory studies conducted on wines before and after HHP processing also confirm that severe treatments can induce changes in the organoleptic properties of wine just after application. Thus, for example, Briones‐Labarca et al. (2017) analyzed several attributes related to appearance, aroma, and taste of young Sauvignon Blanc wine, pressure treated or not, and found that the panelists’ scores were hardly affected after pressure treatments at 300 MPa for 5–15 min. By contrast, higher pressures induced sensory changes, and, thus, the panelists perceived less intense fruity odor and taste and greater aromatic defects in wines treated at 400–500 MPa for 5–15 min. Similar results were reported in red wines. Thus, Mok et al. (2006) observed no significant differences in the scores assigned by panelists to the aroma, taste, mouthfeel, or global sensory quality of red wines before and after treatment at 350 MPa for 10 min, but Tao et al. (2012) detected less intensity in the fruity aroma of red wines treated at 650 MPa for 2 h.

During aging, the organoleptic characteristics of wines are gradually modified due to chemical and enzymatic reactions that take place among the various wine components, especially among the phenolic compounds. In‐line with what was observed in Section 3.3, many authors have noted that the sensorial attributes of pressurized wines evolve more rapidly during storage, thus acquiring the typical characteristics of aged wines sooner, and, depending on the severity of the pressure treatment, these changes may be detectable after more or less months. Moreover, as occurs in conventionally aged wines, the presence and concentration of SO_2_ have a significant effect on the evolution of the organoleptic characteristics of pressurized wines during bottle storage (Christofi et al. 2021, 2020; Lukić et al. 2020).

In general, the color of red wines tends to change from intense red‐purple to lighter red‐orange tones during aging, whereas the color of white wines usually evolves from pale yellow to browner tones, thus losing luminosity. Santos et al. (2013) and Santos, Nunes, Rocha et al. (2013) observed color evolution in both red and white wines, treated at either 425 or 500 MPa for 5 min, during 1 year of bottle storage, at 10–15°C, in the absence of light. They found that color changes in pressurized wines were similar to those in untreated wines, but they occurred more rapidly. Thus, in all red wines, the color parameters a* (red‐green), b* (yellow‐blue), and L* (luminosity) gradually increased during storage, whereas in all white wines, the parameters a* and b* increased, whereas L* decreased. During the first 6 months of storage, differences between untreated and pressurized wines were small, especially for red wines, but, after 12 months, color changes were significantly greater and perceptible to the naked eye in all HHP processed wines. Thus, pressurized red wines showed a more pronounced orange‐red hue than control wines, whereas white wines were significantly more brownish.

Sensory studies, after several months of bottle storage, confirm that HHP processing imparts aged‐like characteristics to wines, and the more severe the treatment, the more intense these characteristics are (Santos et al. 2013, 2016). Thus, after 9 months of bottle storage, Santos et al. (2013) compared the sensorial attributes of untreated red wines, with or without 40 mg L^−1^ of added SO_2_, with SO_2_‐free wines pressurized for 5 min at either 425 or 500 MPa. They found that, confirming the instrumental color measurements, the panelists perceived greater limpidity and browner tones in the pressurized wines, which were also less purple than the untreated ones (both with or without added SO_2_). The aroma and taste of the SO_2_‐free wine treated at 425 MPa were very similar to that of the untreated SO_2_‐added wine, whereas the wines pressurized at 500 MPa showed more intense scents of cooked fruits and spices. These observations are similar to those described by Santos et al. (2016) in pressurized SO_2_‐treated red wines (34 mg L^−1^ of free SO_2_). Thus, after 5 months of bottle storage, wines pressurized at either 500 MPa for 5 min or 600 MPa for 20 min were perceived to have more intense scents of cooked fruit, sulfur aroma, and metallic notes, as well as less fruity flavors, compared to untreated wines. In addition, the authors reported that, at the most severe pressure conditions, differences with control wine increased. Thus, they observed that wine treated at 600 MPa for 20 min, unlike that treated at 500 MPa for 5 min, had greater persistence and bitterness than the control wine as well as less astringency. Despite this, and even though pressurized wines scored lower in terms of aroma, the overall quality of all the wines, pressurized or not, was judged to be similar.

Studies carried out on white wines lead to similar conclusions, although the effects of pressure on their organoleptic characteristics are usually more pronounced than on red wines (Lukić et al. 2020; Santos, Nunes, Rocha, et al. 2013). Thus, Santos, Nunes, Rocha et al. (2013) observed that, after 9 months of storage, free‐SO_2_ white wines pressurized for 5 min at either 425 or 500 MPa were less green and had greater limpidity and browner tones than untreated wines (with or without 40 mg L^−1^ of added SO_2_). In addition, as observed in red wines, pressurized white wines presented cooked fruit scents, probably related to a greater presence of Maillard compounds, less floral and fruity aromas, slightly more bitterness, and less body and balance than untreated wines. As a result, the overall quality scores assigned by the panelists to the pressurized wines were significantly lower. In fact, Santos, Nunes, Rocha et al. (2013) concluded that pressurized white wines were not suitable for marketing as table wines because they exhibited characteristics typical of aged or heat‐treated wines.

Several authors have also shown that, as in untreated wines, the presence and concentration of SO_2_ in pressurized wines largely determine the evolution of their organoleptic characteristics during storage. In general, the higher the SO_2_ content, the better the scores given by the tasting panel. Thus, for example, Christofi et al. (2020) observed that HHP processing of red wines, at 350 MPa for 10 min, weakened the intensity of the red fruit aroma after 12 months of storage. In addition, fruit jam and spicy odors, as well as body and balance, were enhanced, regardless of SO_2_ content. Pressurized wines containing < 60 mg L^−1^ of SO_2_ were perceived as less aromatic and astringent, more oxidized, and with stronger dried fruit aroma than untreated wines, but these differences disappeared when SO_2_ increased up to 100 mg L^−1^.

As mentioned above, several authors have investigated the possibility of combining HHP processing with GSH addition to reduce the required SO_2_ doses in wine. In this sense, Lukić et al. (2020) observed that red and white wines with low SO_2_ content (10 and 25 mg L^−1^ of free SO_2_ in red and white wine, respectively), pressurized at 200 MPa for 5 min and stored for 3, 6, and 12 months, were rated worse by a taste panel than those with higher SO_2_ content (25 and 45 mg L^−1^ of free SO_2_ in red and white wine, respectively). However, when combined with 20 mg L^−1^ of GSH, the scores obtained were similar. These results were confirmed by Christofi et al. (2021), who tested the effect of HHP processing combined with different doses of SO_2_ (0–100 mg L^−1^) and 10 mg L^−1^ of GSH on red wine after 12 months of storage. They found that HHP processing, at 400 MPa for 5 min, in the presence of GSH allowed the SO_2_ dose to be reduced to 40–60 mg L^−1^. Pressurized samples containing 40 and 60 mg L^−1^ of SO_2_ were perceived as less astringent and more balanced, as well as having a better overall quality.

Several studies in the literature compare organoleptic characteristics of pressure‐aged wines with those of wines conventionally aged, either in wooden barrels or in steel vats with wood chips added (Santos et al. 2019; Tao et al. 2016; Valdés et al. 2021). Thus, for example, Santos et al. (2019) compared the sensory attributes of red wines treated at 500 MPa for 5 min and stored in polyethylene bottles for 5 months with those of wines aged for 3 months, either in oak barrels or in steel tanks with oak chips, micro‐oxygenated or not, and then stored in polyethylene bottles for 2 months. They reported that the panelists did not perceive any color differences between the wines aged by different techniques, but significant divergences were detected in the aroma. Thus, wines aged in contact with wood (barrels or wood chips) exhibited more intense woody, toasty, and spicy notes than pressurized wines as expected, but no significant differences were perceived in taste attributes such as astringency, acidity, bitterness, persistence, body, or sweetness. In fact, all wines, regardless of the aging technique used, received similar scores in global quality, differing only in aroma, which was obviously superior in the wood‐aged wines. When pressure aging is performed with wood chips immersed in the wine, the organoleptic characteristics seem to be similar to those of conventionally aged wines (Tao et al. 2016; Valdés et al. 2021). Thus, Valdés et al. (2021) reported that, after 3 months of bottle storage, triangular discriminatory tests revealed no differences between red and white wines conventionally macerated with holm oak chips in steel tanks for 45 days and those macerated at 400 MPa for either 5 or 30 min. White wines macerated in holm oak chips, either conventionally or under pressure, had a deep yellow color with slight golden hues and sawdust and toasty scents, and they were defined by the panelists as more complex and astringent than non‐macerated wines. By contrast, the panelists could not distinguish between red wines macerated with holm oak chips, either conventionally or under pressure, and those that were not macerated. This is probably because red wines are more complex than white wines, and, therefore, they require longer maceration times than those tested by Valdés et al. (2021) to achieve noticeable organoleptic changes.

## 5 Challenges and Future Perspectives

**FIGURE 4:**
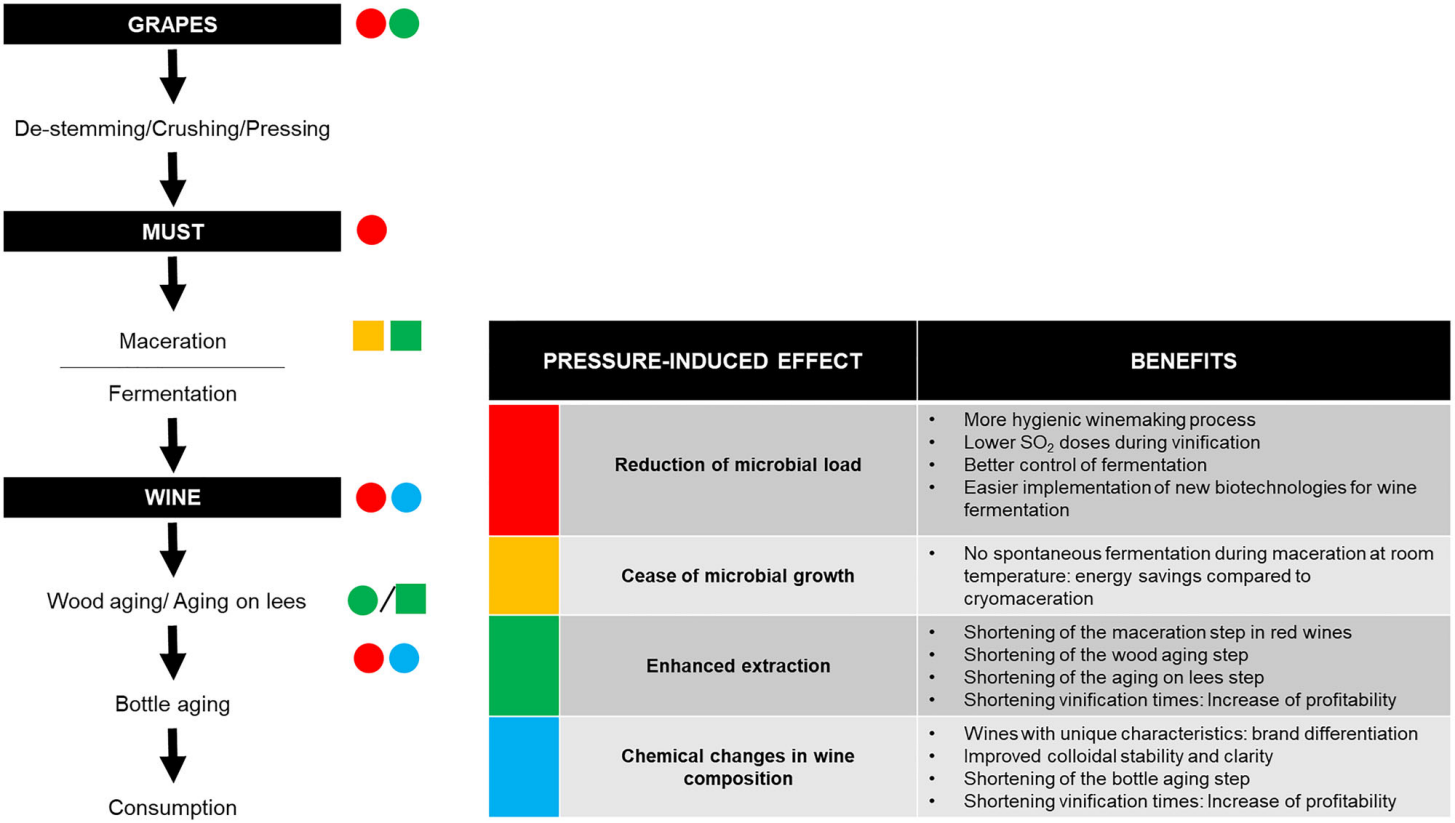
Steps of the winemaking process in which high hydrostatic pressure could be applied either to grapes, must, or wine; pursued effects; and potential benefits. Circles represent HHP processing for some minutes, whereas squares represent steps carried out entirely under pressure.

The foregoing sections reveal the enormous potential of HHP processing to improve various stages of the winemaking process. As shown in Figure 4, HHP treatments could be applied to either grapes, must, or wine, and, depending on the desired effect, industrial implementation would be a more or less complicated challenge.

**The pressurization of grapes** would reduce their microbial load, thus allowing a more hygienic winemaking process. As already mentioned, this would also reduce the amount of SO_2_ required during vinification, which is currently of great interest to the industry. In addition, the elimination of the indigenous grape microbiota would facilitate the development of new fermentation biotechnologies because, as detailed above, it would favor the implantation of non‐*Saccharomyces* yeasts or the co‐inoculation of yeasts and malolactic bacteria, among others. Finally, grape pressurization would also favor the extraction of phenolic components from skins and seeds, thus accelerating the maceration phase of red wines as commented in Section 3.2.1.

**FIGURE 5:**
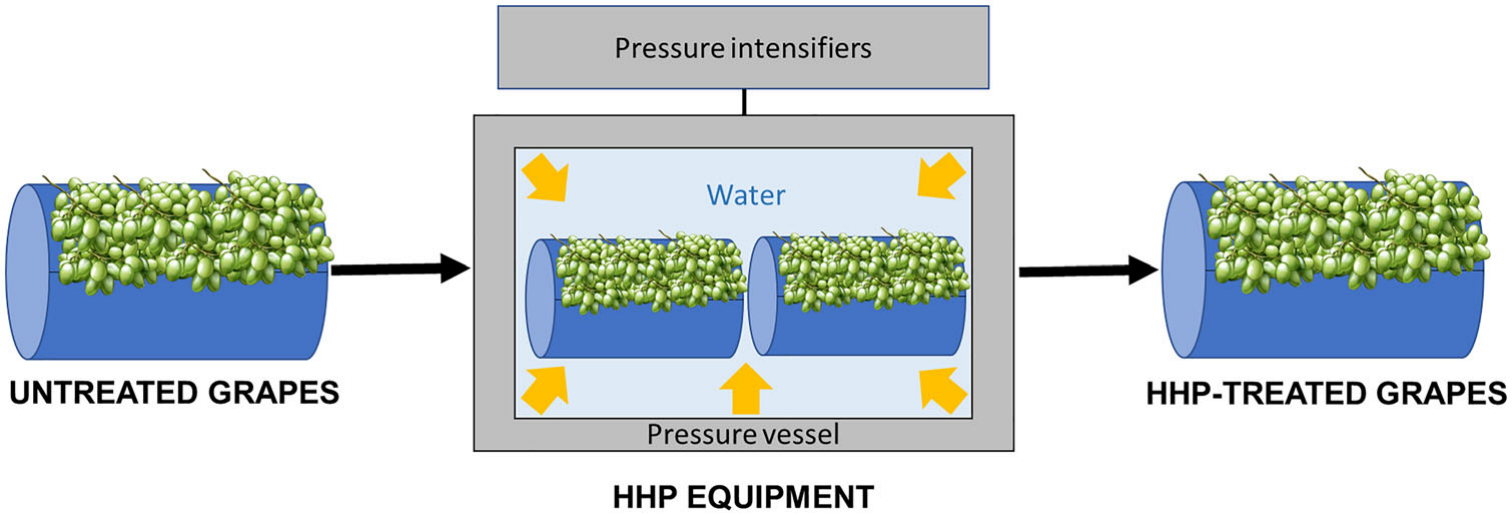
Schematic view of an imaginary industrial implementation of HHP processing of grapes. The grapes would be directly placed, without packing, in baskets, and these would then be fed into the HHP equipment for treatment. Current commercial HHP equipment could be used. HHP, high hydrostatic pressure.

The industrial pressurization of grapes would not require the development of new HHP equipment adapted to this use, but existing equipment could be used. As is currently done for crustaceans and bivalves, the grapes would be directly placed, without packaging, in baskets and these would then be fed into the HHP equipment for treatment (Figure 5). Since the grapes would be treated unpackaged, only water should be used as pressurizing fluid to avoid undesired interactions between the grapes and other fluids. In addition, if reducing the microbial load is the primary effect sought, the treated grapes should then be quickly processed under the most hygienic conditions possible since, being unpacked, they are susceptible to recontamination after treatment.

**Must and wine pressurization** would also provide various benefits in the winemaking process as shown in Figure 4. For example, pressurizing must would reduce its microbial load, making it easier for the oenologist to control fermentation (Delfini et al. 1995; Tonello et al. 1998). On the other hand, wine pressurization could be carried out for many different purposes: to reduce the risk of microbial spoilage as well as the doses of SO_2_ to be used (Christofi et al. 2020; Santos et al. 2012; Silva and van Wyk 2021; van Wyk et al. 2018), to give the wine unique organoleptic characteristics positively perceived by the consumer (Christofi et al. 2021; Morata et al. 2015; Santos et al. 2013), to favor its colloidal stability and clarity (Tabilo‐Munizaga et al. 2014), or to accelerate the typical reactions that occur during aging, thus reducing aging time (Tao et al. 2016; Valdés et al. 2021). Furthermore, when wines are pressure treated together with wood chips or lees, the desired effect would be to enhance the extraction of various compounds from the wood or lees into the wine, thus accelerating the aging process.

**FIGURE 6:**
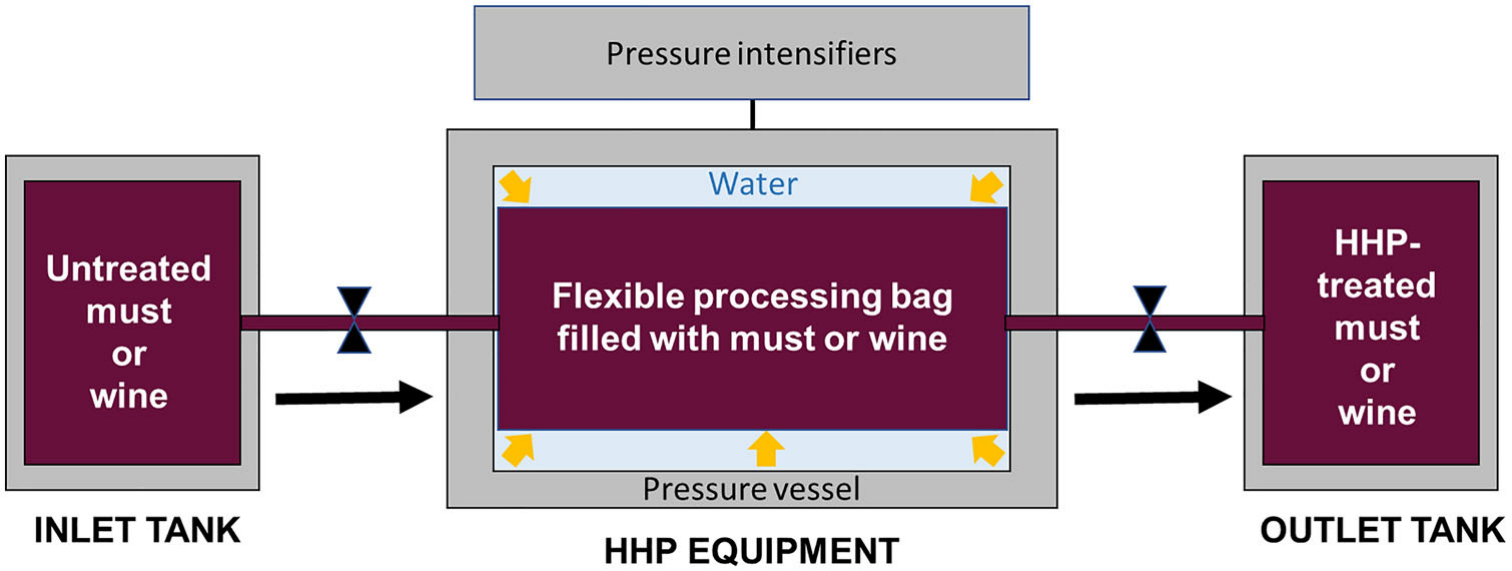
Schematic view of an imaginary industrial implementation of HHP processing of musts and wines. Must or wine would be pumped from the inlet tank into a flexible processing bag inside the pressure vessel where they would be pressure treated. Current commercial in‐bulk HHP equipment, specially designed for the treatment of pumpable liquids, would be required. HHP, high hydrostatic pressure.

The industrial pressurization of musts and wines would, in principle, require the use of HHP equipment specially designed for the treatment of pumpable liquids. Although not yet widely used in the beverage industry, there are different types on the market, being the “in‐bulk HHP equipment,” offered by the Spanish company Hiperbaric, being one of the most demanded (Hiperbaric 2020a). The design of this equipment includes a system of inlet and outlet tanks where must or wine would be pumped before and after HHP processing and a flexible processing bag, inside the pressure vessel, where they would be pressure treated (Figure 6). After processing, the must would undergo vinification process, whereas the wine, depending on the point in the process at which pressure was applied and the type of vinification, could even be bottled. To do so, the treated wine would be pumped from the outlet tank to an ultra‐clean filling line where it could be packaged in glass bottles. Of course, pressurization of the wine at the end of the winemaking process could also be done in conventional “in‐pack HHP equipment,” but since rigid glass bottles do not transmit pressure, this would require flexible packaging such as plastic bottles or pouches. However, plastic packaging could have a negative impact on the acceptance of wine by consumers, who are used to traditional glass bottles and increasingly aware of the importance of reducing plastic consumption due to its harmful effects on the environment. Moreover, many authors in the literature have shown that plastic containers in direct contact with wine can adsorb aroma compounds, and, depending on their oxygen barrier properties, significant oxidation can occur during storage, resulting in a significant deterioration of the wine taste (Ghidossi et al. 2012; Moreira et al. 2016; Revi et al. 2014). Furthermore, Puig et al. (2003) reported that, after treating white and red wines in plastic containers at 400–500 MPa for 5–15 min, panelists detected plastic aromas and flavors in the wine. To the best of our knowledge, no further studies have been carried out since then on the transfer of odors and flavors from plastic containers to wine during HPP processing, but it is obvious that this could be a major problem if “in‐pack HHP equipment” is to be used.

Industrial pressurization of wine together with wood chips would, however, be a challenge today. Chips would pose a problem for HHP equipment designed to treat pumpable liquids, and it would be necessary to design a solution specifically adapted to this process.

Even though, since 2019, the International Organization of Vine and Wine authorized HHP processing of grapes and musts (OIV 2024a), this technology has not yet been implemented in the wine industry. This may be due to different reasons, including a lack of an innovative mindset or the limited number of scientific studies supporting certain applications, among others. However, one of the most important obstacles is, undoubtedly, the high cost of HHP equipment. High pressure processing involves large and expensive equipment, costing between $500,000 and $4000,000 depending on capacity and degree of automation (Janahar and Balasubramaniam 2022). To evaluate the economic sustainability of HHP, it is important to consider not only the acquisition cost of the HHP machine but also the total initial investment required, the operation and maintenance costs during the use of the equipment, and the end‐of‐life costs at the time of disposal. In this context, Cacace et al. (2020) used “life cycle costing” (LCC) methodology to conduct a detailed economic analysis of HHP processing in a plant capable of processing 270 kg of bottled orange juice per cycle. Among the initial investment costs, they included the price of the equipment, the costs of transport, installation, and testing, and those of fitting out a suitable building to house the HHP machine with connections to a power source. The main operation costs identified for the HHP plant were those derived from energy and water consumption, lubricants for the pressure intensifiers, manpower, and packaging of the processed product. Other minor operating costs included safety costs (gloves, masks, safety shoes, and work clothes), cleaning and sanitization costs (professional detergents), insurance, and other annual fees. Maintenance costs included those derived from preventive, scheduled, and reactive maintenance, whereas end‐of‐life costs were considered to be the residual value of the equipment after a given lifetime, that is, a benefit. Considering a 20‐year lifecycle, a production of 2.7 × 10^6 ^kg of juice per year, and an interest rate of 0.0096, Cacace et al. (2020) estimated the unit cost of the HHP treatment to be 0.1853€/kg of juice. They found that the operation costs accounted for the highest proportion of the LCC (71.2%), whereas the initial investment and maintenance costs accounted for only 18.0% and 10.8%, respectively. Furthermore, within the operation costs, those corresponding to packaging material required for juice processing, energy consumption, and the salaries of the employees were the most important, representing 49.0%, 20.8%, and 17.9% of the total operating costs, respectively. The unit costs calculated by Cacace et al. (2020) are similar to those estimated by other authors in the literature (Aganovic et al. 2021; Sampedro et al. 2014) and seem to be quite affordable for a juice industry. However, it is important to note that the calculations were based on an annual production of 2.7 × 10^6 ^kg of juice per year. The annual production of most wineries is significantly lower and seasonal in nature, and, therefore, the unit cost for wine production can considerably increase. For example, for a winery with an annual production of 300,000 L/year, unit costs would be higher than 0.8€/L, even after considering only half of the initial investment, operation, and maintenance costs estimated by Cacace et al. (2020). Moreover, as most wineries are small‐ and medium‐sized companies, the initial investment required to purchase even the cheapest HHP equipment may be prohibitive. Nevertheless, a solution to this problem may be the high‐pressure processing service or tolling offered by some companies. This service allows small‐ and medium‐sized food companies to pay for the processing of their products without having to purchase an HHP machine (Hiperbaric 2024; Yamamoto 2017). Tolling, which is currently available in many countries, would allow wineries to process grapes, must, or wine without initial investment in equipment or maintenance costs. In this way, oenologists could test, without risk, the benefits of HHP processing on the quality of their wines and check consumer acceptance before assessing the profitability of purchasing their own equipment.

Although this review reveals that many of the interesting opportunities that HHPs offer in winemaking have already been identified and even tested, many other possibilities still remain unexplored. For example, many papers demonstrate that pressure at sublethal levels acts as an effective inhibitor of microbial growth. Thus, foods can be preserved at pressures of 50–100 MPa at room temperature, known as hyperbaric storage, for long periods of time, even up to years in certain cases (Bermejo‐Prada et al. 2016; Lemos et al. 2020; Otero and Pérez‐Mateos 2021; Santos et al. 2020). These relatively low pressure levels can still cause significant physical and chemical modifications in food matrices, with some physical, chemical, and biochemical reactions either hindered or enhanced (Bermejo‐Prada et al. 2015; Bermejo‐Prada and Otero 2015; Shkolnikov et al. 2020). All these pressure‐induced effects could open the possibility of improving and/or accelerating some specific steps of the winemaking process by conducting these steps under these moderate pressures.

Because pressure can stop microbial growth, maceration under pressure could not only improve the extraction of pigments, tannins, and aromatic molecules from the grape skins but also prevent spontaneous fermentations, thus allowing an effective separation of the maceration and fermentation steps (Figure 4). Moreover, unlike conventional cryomaceration, it could be conducted at room temperature, resulting in considerable energy savings. Furthermore, because sublethal pressure work acts as an on/off switch to stop/start fermentation (Mota et al. 2015), musts macerated under sublethal pressure could either be subsequently fermented by the native grape microbiota or processed to reduce/eliminate the wild microflora, depending on the specific interests of the oenologist.

Wine aging under moderate pressure is another unexplored possibility (Figure 4). Pressure, like other storage conditions, such as temperature, humidity, or light exposure, could significantly affect the complex chemical reactions that occur during wine aging. In this sense, the aging of bottled wines underwater, usually in the sea but sometimes in springs, has recently attracted the attention of both wineries and consumers who are willing to pay more for these wines. Underwater‐aged wines are said to have more definition and character than conventionally aged wines, and these differences are theoretically attributed to the underwater storage conditions (pressure, temperature, absence of light, and anaerobic conditions). Some recent scientific studies confirm differences in the chemical composition of red and white wines aged either in a cellar or underwater, at a depth of 20–25 m, that is, at a pressure of about 0.3 MPa (Birkić et al. 2024; Mercanti et al. 2024). For example, Mercanti et al. (2024) found that Merlot and Sangiovese red wines aged at 25 m below sea level had higher concentrations of TPs and anthocyanins and lower levels of non‐flavonoid compounds than those conventionally aged in a cellar. In addition, Birkić et al. (2024) detected significantly higher concentrations of naringenin and myricetin in Malvasia white wines aged underwater in springs in the Northern Adriatic than in those conventionally aged. However, as temperature, oxygen conditions, or light exposure were not identical in conventional and underwater aging, these differences should not be only attributed to pressure, and further studies are needed to evaluate the role of pressure in wine aging.

**FIGURE 7:**
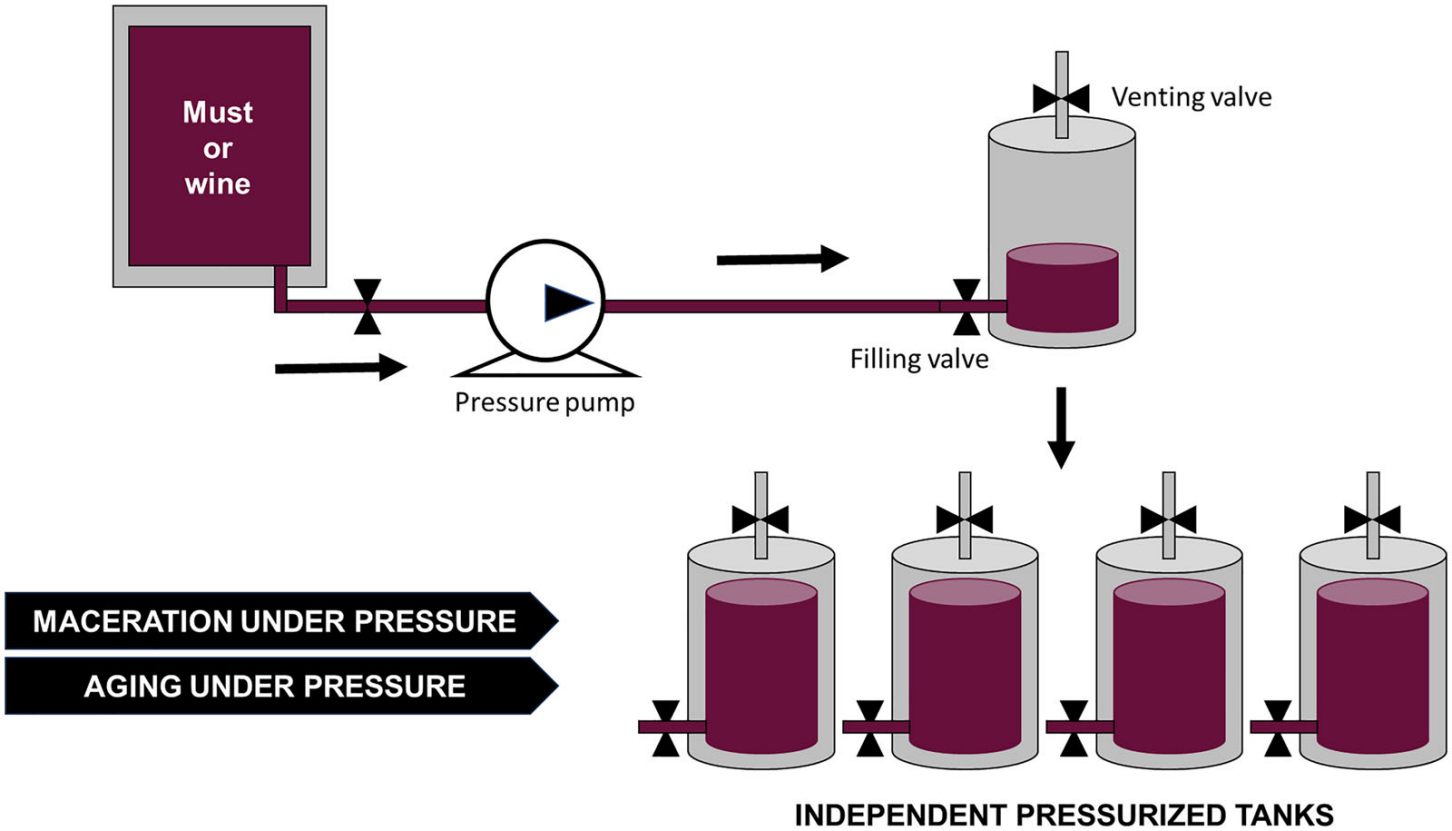
Schematic view of an ideal industrial implementation of either must maceration or wine aging under pressure.

The industrial implementation of maceration and aging under moderate pressure could be relatively simple and would require the design and development of equipment similar to that required for hyperbaric storage (Figure 7). Thus, it would consist of a hydraulic pump and a set of tanks that can be pressurized, made independent from the pump, and kept pressurized for relatively long periods of time, from hours to several weeks or months (Bermejo‐Prada et al. 2017). Obviously, the size and weight of these tanks will depend on the working pressure, and, therefore, this pressure will determine the industrial feasibility of these innovative processes.

All of the above‐described potential advantages of HHP offer exciting opportunities that need to be explored and evaluated. Thus, further research is needed to fully elucidate the benefits of HHP in winemaking, to understand the mechanisms involved, to optimize process parameters depending on the desired effects, or to design solutions for industrial implementation.

## 6 Conclusions

All the information analyzed in this review reveals that HHP can effectively help to address many of the major challenges facing the wine industry today. On the one hand, HHP processing can reduce the microbial load in grapes, must, and wine, thus reducing the doses of SO_2_ required during vinification and satisfying the current consumer demand for wines with low SO_2_ content. Total replacement of SO_2_ by HHP processing to obtain more natural wines, free of allergens, and suitable for all types of consumers, does not seem feasible because the antioxidant and antioxidasic properties of SO_2_ are not provided by pressure. Nevertheless, the combination of HHP processing with alternative products to SO_2_ that provide antioxidant and antioxidasic protection to wine, such as GSH, chitosan, or ascorbic acid, may be a plausible strategy to explore in the future. On the other hand, pressure‐induced microbial inactivation in grapes and musts also facilitates the implementation of new biotechnologies for wine fermentation to obtain high‐quality innovative wines with their own distinctive characteristics, thus mitigating the negative effects of climate change or the use of commercial starters on wine quality. In this sense, pressure‐induced chemical changes in wine composition can also contribute to the production of wines with unique characteristics that attract consumer attention in a competitive global market.

Furthermore, HHP capacity to rupture cell walls and enhance permeability allows for the efficient extraction of pigments, tannins, and aromatic molecules from grape skins, improving both the color and antioxidant capacity of the final product. This enhanced extraction shortens maceration times and promotes superior aging potential, resulting in wines of greater complexity and richness. When used during aging with oak chips or on lees, HHP promotes the extraction of wood‐ or lees‐related compounds and facilitates desirable chemical transformations, cutting down the time required for traditional aging. Thus, by accelerating maceration and aging steps, HHP can reduce the risk of microbial spoilage associated with these steps, shorten vinification times, and increase the profitability of wine production. Other unexplored applications of high pressure, such as maceration and wine aging under moderate pressure, require further research to determine their true potential to accelerate or improve winemaking.

Industrial implementation of HHP processing of grapes, must, and wine is readily achievable as suitable equipment is commercially available. However, several challenges must be addressed to promote its adoption by the wine industry. HHP equipment involves significant capital investment, and the seasonal nature of the wine production can make the economic sustainability of HHP processing difficult for small‐ and medium‐sized wineries. In this context, HHP tolling can be a suitable solution to access the technology on an as‐needed basis without the need to purchase and maintain expensive HHP equipment. Moreover, cooperative models where multiple wineries share HHP equipment could reduce individual investment burdens. On the other hand, it is important to note that pressures of 400 MPa have been found to be enough to both dramatically decrease wild yeast population in grapes and enhance solid–liquid extraction processes. Therefore, HHP solutions for facilitating the implementation of new biotechnologies for wine fermentation or shortening maceration and aging steps could be carried out in cheaper HHP equipment, able to work at a maximum of 400 MPa, than standard 600‐MPa devices used in other food industries. This can also contribute to reduce energy consumption and maintenance costs.

Energy consumption is another critical concern as it accounts for a significant part of the total operating costs. Implementing energy‐efficient hydraulic systems and energy recovery technologies and focusing on low‐pressure applications for certain winemaking steps could significantly reduce operating costs and environmental impact.

Commercialization also presents significant challenges. Although, thanks to the growing presence of HHP in the food industry, many consumers are already familiar with this technology, its use in such a tradition‐bound product as wine may cause some consumers to reject it. In this regard, consumer concerns about the effects of HHP on wine authenticity and quality should be dispelled through education and branding efforts that highlight the benefits of HHP in reducing the use of chemical additives, conferring unique quality characteristics to wine, and promoting environmentally friendly production processes.

Despite these challenges, innovative solutions and collaboration among HHP equipment manufacturers, researchers, and wineries can pave the way for HHP to become a reality in the wine industry, improving quality, innovation, and sustainability.

## Author Contributions

**Laura Otero**: conceptualization, investigation, supervision, writing–original draft, writing–review and editing, visualization. **Lucía del Prado**: investigation, visualization, writing–original draft, writing–review and editing. **Antonio Morata**: funding acquisition, project administration, writing–review and editing.

## Conflicts of Interest

The authors declare no conflicts of interest.
